# Neutrophil-tropic APL@Mn_3_O_4_ nanozymes alleviate myocardial ischemia-reperfusion injury through redox regulation

**DOI:** 10.1016/j.mtbio.2026.103815

**Published:** 2026-11-04

**Authors:** Fu-hang Wang, Yuan-song Zhuang, Jun-jie Ma, Yu-hang Li, Quan-xu An, De-liang Shen

**Affiliations:** a Department of Cardiology, The First Affiliated Hospital of Zhengzhou University, Zhengzhou, 450052, China; b Department of Cardiology, Zhumadian Central Hospital, Zhumadian, 463000, China; c Department of Cardiology, Zhoukou Central Hospital, Zhoukou, 466000, China

**Keywords:** Neutrophil tropism, Manganese oxide nanozymes, Myocardial ischemia-reperfusion injury, Oxidative stress, Inflammation

## Abstract

Myocardial ischemia-reperfusion injury (MIRI) is driven by coupled oxidative and inflammatory amplification after reperfusion, with activated neutrophils acting as early upstream effectors that propagate secondary cardiomyocyte injury. Strategies limited to downstream oxidative damage may therefore be insufficient to interrupt this reinforcing cascade. We developed an agglutinated-protein liposome formulation encapsulating Mn_3_O_4_ nanozymes (APL@Mn_3_O_4_), designed to integrate delivery directed toward activated neutrophils with catalytic redox regulation. The agglutinated-protein liposomal surface supported complement-associated tropism toward activated neutrophils under inflammatory conditions, whereas the Mn_3_O_4_ core retained catalytic antioxidant activity for reactive oxygen species (ROS) regulation. *In vitro*, APL@Mn_3_O_4_ reduced neutrophil oxidative and inflammatory responses without measurably impairing migratory capacity under the tested conditions. In a neutrophil-cardiomyocyte co-culture system, APL@Mn_3_O_4_ attenuated oxidative stress and apoptosis in injured cardiomyocytes. *In vivo*, APL@Mn_3_O_4_ preferentially associated with circulating neutrophils, attenuated oxidative and inflammatory myocardial injury, and improved post-reperfusion cardiac remodeling and function. These findings support APL@Mn_3_O_4_ as a neutrophil-tropic nanozyme platform for alleviating MIRI by attenuating pathological neutrophil-associated oxidative and inflammatory activity and reducing secondary cardiomyocyte injury.

## Highlights

• Complement-mediated recognition enhances APL@Mn_3_O_4_ association with neutrophils.
• Neutrophils enhance APL@Mn_3_O_4_ localization after myocardial ischemia–reperfusion.
• Mn_3_O_4_ nanozyme suppresses neutrophil reactive oxygen species and cytokine release.
• APL@Mn_3_O_4_ limits myocardial injury and preserves cardiac function.
• Hematology, cytokine, histology, and tissue manganese data support tolerability.

## Graphical abstract

**Figure:**
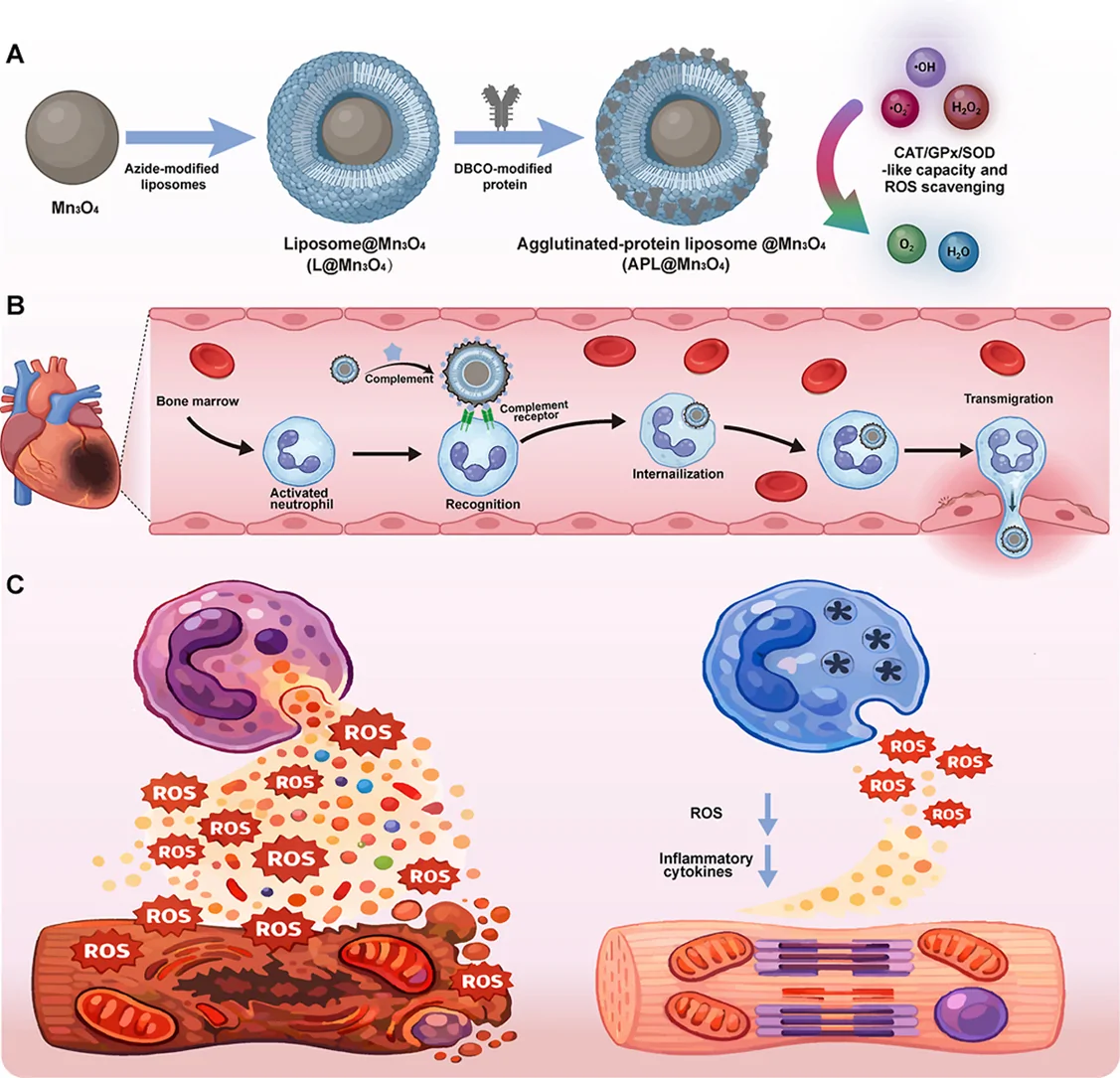

## 1 Introduction

Although timely reperfusion is essential for salvaging ischemic myocardium, myocardial ischemia-reperfusion injury (MIRI), driven largely by excessive reactive oxygen species (ROS) accumulation and associated inflammatory amplification, remains a major limitation of reperfusion therapy [1], [2]. MIRI is therefore better understood not as a brief ROS surge alone, but as an evolving oxidative-inflammatory process in which oxidative damage and inflammatory activation reinforce each other [2], [3]. Neutrophils, among the earliest inflammatory cells recruited after reperfusion, are a prominent source of pathological ROS and further propagate myocardial damage through proteases and pro-inflammatory mediators, placing them at an upstream interface between oxidative injury and inflammatory activation [4], [5], [6]. Within this oxidative-inflammatory milieu, cardiomyocytes are particularly vulnerable to oxidative stress-induced mitochondrial dysfunction and apoptotic death [7], [8]. Preferentially directing antioxidant intervention toward activated neutrophils may therefore help limit pathological ROS accumulation and the associated inflammatory amplification, thereby reducing secondary myocardial injury after reperfusion [4], [5], [6].

Given the central role of oxidative stress in MIRI, antioxidant intervention has long been considered a rational therapeutic strategy [2], [9]. However, conventional antioxidant strategies—including vitamins C and E, the free-radical scavenger edaravone, and the glutathione precursor N-acetylcysteine—have shown limited or inconsistent clinical efficacy [9]. Their translation is constrained by rapid clearance, inadequate delivery to the injured myocardium, and a limited ability to address the dynamic ROS response arising from multiple sources [9], [10], [11]. These limitations have prompted interest in nanozymes based on metal oxides, noble metals, and Prussian blue, which have shown potential for catalytic ROS elimination and sustained redox regulation [12], [13]. However, limited ROS-scavenging modalities may not adequately address the complex redox disturbance in MIRI; peroxidase-like or oxidase-like pathways may carry pro-oxidant liability, and the long-term safety of noble-metal or non-physiological cores remains uncertain [12], [13], [14]. These considerations favor nanozyme systems combining broad-spectrum antioxidant catalysis, favorable biocompatibility, and compatibility with functional delivery platforms. Mn_3_O_4_ is well suited to these requirements because its superoxide dismutase (SOD)-, catalase (CAT)-, and glutathione peroxidase (GPx)-like activities support sustained, multimodal redox regulation, while manganese, an essential trace element, provides a physiologically relevant elemental basis [13], [15]. Accordingly, we selected Mn_3_O_4_ as the catalytic core of a functional nanozyme platform for alleviating MIRI.

Despite its catalytic potential, unmodified Mn_3_O_4_ lacks engineered cell-selective targeting, potentially limiting its delivery to activated neutrophils. Given the upstream role of these cells in early ROS amplification and associated inflammatory propagation in MIRI, preferentially directing antioxidant activity toward this population may be pathophysiologically more relevant than untargeted ROS scavenging [4], [6], [16], [17]. Previous work showed that liposomes bearing an agglutinated protein (AP) surface architecture were preferentially taken up by neutrophils under inflammatory conditions and that this behavior depended on complement opsonization, with complement component 3 (C3) among the major adsorbed complement proteins [18]. Related studies further support both the contribution of complement opsonization to nanoparticle uptake by neutrophils and other leukocytes [19], [20] and the feasibility of neutrophil-mediated liposome delivery to inflammatory or ischemic lesions [21]. Together, these findings provide a rationale for engineering AP-functionalized liposomes encapsulating Mn_3_O_4_ nanozymes to promote preferential association with activated neutrophils, with complement-associated recognition as a potential contributing mechanism.

We therefore constructed AP-functionalized liposomes encapsulating Mn_3_O_4_ nanozymes (APL@Mn_3_O_4_; Scheme 1). We hypothesized that AP functionalization would promote preferential association of the formulation with activated neutrophils and direct the catalytic ROS-scavenging activity of Mn_3_O_4_ toward this cell population, thereby limiting pathological ROS accumulation and the associated inflammatory amplification, ultimately reducing secondary myocardial injury. To test this hypothesis, we characterized the physicochemical and catalytic properties of APL@Mn_3_O_4_, examined its association with activated neutrophils and its effects on neutrophil function and cardiomyocyte injury *in vitro*, and assessed its biodistribution and effects on acute myocardial injury and post-injury cardiac remodeling *in vivo*.

**Scheme 1:**
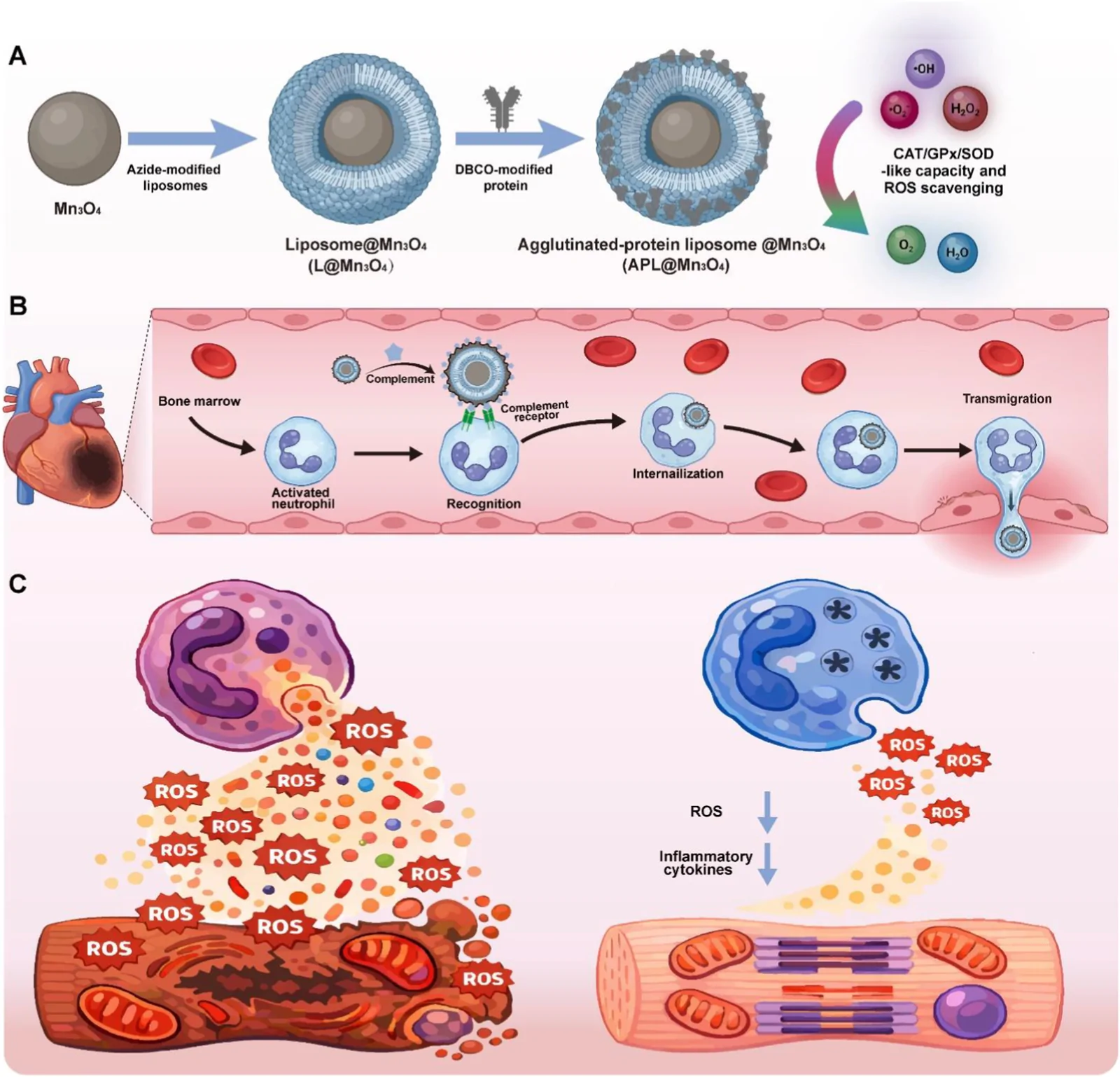
**Schematic illustration of the construction of APL@Mn_3_O_4_ and its proposed mechanism for alleviating myocardial ischemia-reperfusion injury (MIRI).** (A) Construction of APL@Mn_3_O_4_. Mn_3_O_4_ nanozymes were first encapsulated into liposomes to form L@Mn_3_O_4_, followed by agglutinated protein (AP) functionalization to generate APL@Mn_3_O_4_ (an agglutinated-protein liposome formulation encapsulating Mn_3_O_4_ nanozymes). (B) Proposed neutrophil-associated delivery process after intravenous administration. Under inflammatory conditions, APL@Mn_3_O_4_ undergoes complement-associated recognition and preferentially associates with circulating neutrophils, consistent with a neutrophil-associated component of myocardial delivery after reperfusion. (C) Proposed therapeutic mechanism in MIRI. By modulating pathological neutrophil responses, APL@Mn_3_O_4_ attenuates pathological neutrophil-associated oxidative and inflammatory output. These effects reduce secondary cardiomyocyte oxidative stress, mitochondrial dysfunction, and apoptosis, thereby alleviating acute myocardial injury and supporting improved post-reperfusion cardiac remodeling.

## 2 Materials and methods

### 2.1 Materials

1,2-Dipalmitoyl-sn-glycero-3-phosphocholine (DPPC), cholesterol, and 1,2-distearoyl-sn-glycero-3-phosphoethanolamine-N-[azido(polyethylene glycol)-2000] (azide-PEG2000-DSPE) were purchased from Shanghai Aladdin Biochemical Technology Co., Ltd. (Shanghai, China). Mn(OAc)_2_·4H_2_O, N,N-dimethylformamide (DMF), 2,3,5-triphenyltetrazolium chloride (TTC), Evans blue, fetal bovine serum (FBS), recombinant murine tumor necrosis factor-α (TNF-α), N-formyl-Met-Leu-Phe (fMLP), cobra venom factor, the lipophilic carbocyanine dye DiD, 2′,7′-dichlorodihydrofluorescein diacetate (DCFH-DA), dihydroethidium (DHE), rat immunoglobulin G (IgG), dibenzocyclooctyne-N-hydroxysuccinimidyl ester (DBCO-NHS ester), and other analytical-grade reagents were obtained from Sigma-Aldrich (St. Louis, MO, USA). Cell Counting Kit-8 (CCK-8) and assay kits for JC-1 mitochondrial membrane potential, Calcein-AM/EthD-1 Live/Dead viability, TUNEL apoptosis detection, Annexin V-FITC/PI apoptosis detection, SOD-like, CAT-like, and GPx-like activities, hydroxyl radical (•OH)-scavenging capacity, and 2,2′-azino-bis(3-ethylbenzothiazoline-6-sulfonic acid) (ABTS)-based total antioxidant capacity were obtained from Beyotime Biotechnology (Shanghai, China). Primary antibodies against CD86, CD206, CD31, α-smooth muscle actin (α-SMA), and α-actinin were from Abcam (Cambridge, UK). Alexa Fluor-conjugated secondary antibodies were obtained from Proteintech Group, Inc. (Wuhan, China). Antibodies against Ly6G, CD45, CD11b, Ly6C, and F4/80 for flow cytometry were purchased from Elabscience Biotechnology Co., Ltd. (Wuhan, China).

### 2.2 Synthesis of Mn_3_O_4_ nanoparticles (Mn_3_O_4_ NPs)

Mn_3_O_4_ NPs were synthesized via a solvothermal method. Mn(OAc)_2_·4H_2_O (2.45 g) was dissolved in DMF (70 mL) and transferred to a Teflon-lined stainless-steel autoclave. The reaction proceeded at 130 °C for 8 h, followed by natural cooling to room temperature. Nanoparticles were collected by centrifugation (12,000 × g, 10 min), washed three times with ethanol and deionized water, and resuspended in sterile water.

### 2.3 Preparation of L@Mn_3_O_4_ and APL@Mn_3_O_4_

APL@Mn_3_O_4_ was prepared by thin-film hydration and click conjugation. Liposomes comprised DPPC, cholesterol, and azide-PEG2000-DSPE at a molar ratio of 58:40:2. For fluorescence-labeling experiments, DiD was added at a lipid-to-dye mass ratio of 400:1. After dissolution in chloroform, the solvent was evaporated under nitrogen to form a thin lipid film. The film was hydrated with HEPES buffer (100 mM NaCl, 10 mM HEPES, pH 7.4) containing Mn_3_O_4_ NPs, followed by sonication for three cycles of at least 5 min each. The suspension was then extruded through a 100 nm polycarbonate membrane to obtain the liposomal formulation encapsulating Mn_3_O_4_ NPs (L@Mn_3_O_4_). For AP functionalization, rat IgG was reacted with DBCO-NHS ester at a molar ratio of 20:1 for 1 h and purified to remove unreacted DBCO-NHS ester. Purified DBCO-modified IgG was incubated overnight with azide-functionalized L@Mn_3_O_4_ to generate APL@Mn_3_O_4_ via click conjugation. Unbound IgG was removed by size-exclusion chromatography.

### 2.4 Physicochemical characterization

Hydrodynamic size distributions and zeta potentials of bare Mn_3_O_4_ NPs and APL@Mn_3_O_4_ were measured by dynamic light scattering (DLS; Zetasizer Nano ZS, Malvern Instruments). Fresh suspensions were deposited on carbon-coated copper grids, dried, and examined by transmission electron microscopy (TEM; Tecnai G2, FEI). Mn_3_O_4_ X-ray diffraction (XRD) patterns were recorded using a D8 ADVANCE diffractometer (Bruker; Cu Kα; 2θ = 5–90°). X-ray photoelectron spectroscopy (XPS) spectra were acquired using a K-Alpha spectrometer (Thermo Fisher Scientific; monochromated Al Kα), with binding energies calibrated to C 1s at 284.8 eV and peaks fitted using XPSPeak. Fourier-transform infrared (FTIR) spectra of rat IgG, L@Mn_3_O_4_, and APL@Mn_3_O_4_ were recorded over 4000–500 cm^−1^ using a Nicolet iS10 spectrometer (Thermo Fisher Scientific). Unbound IgG collected by size-exclusion chromatography was quantified at 280 nm using a U-3900 ultraviolet–visible spectrophotometer (Hitachi High-Tech Corporation). Conjugation efficiency was calculated as [(IgG input − unbound IgG)/IgG input] × 100%. After size-exclusion separation of the formulation from non-encapsulated Mn_3_O_4_, Mn in the input and non-encapsulated fraction was quantified by inductively coupled plasma mass spectrometry (ICP-MS; Agilent 7900, Agilent Technologies) following nitric-acid digestion. Mn_3_O_4_ encapsulation efficiency was calculated on an Mn-equivalent basis as [(Mn input − non-encapsulated Mn)/Mn input] × 100%. For both nanoparticle preparations, hydrodynamic diameters were monitored in PBS at 4 °C and serum at 37 °C for up to 72 h.

### 2.5 Enzyme-mimetic and antioxidant assays

Catalytic antioxidant activities of Mn_3_O_4_ NPs and APL@Mn_3_O_4_ were assessed by electron spin resonance (ESR) and commercial kits. For ESR, hydroxyl radicals (•OH) generated by UV-irradiated TiO_2_ were trapped with 5,5-dimethyl-1-pyrroline N-oxide (DMPO), whereas superoxide radicals (O_2_•^-^) were generated using xanthine/xanthine oxidase. Scavenging activity was quantified from the nanoparticle-induced decrease in ESR signal intensity. SOD-like, CAT-like, GPx-like, •OH-scavenging, and ABTS-based total antioxidant capacity were measured according to the manufacturer's instructions. Formulations were tested in parallel at matched concentrations and normalized to the corresponding controls.

### 2.6 Cell culture, primary cardiomyocyte isolation, and neutrophil isolation

H9c2 cells were cultured in DMEM containing 10% FBS at 37 °C and 5% CO_2_. Primary neonatal rat cardiomyocytes (NRCMs) were isolated from 1-day-old Sprague–Dawley rats and identified by sarcomeric α-actinin staining. All animal procedures were performed under the approved animal protocol (Approval No. ZZU-LAC20210903(02)); detailed procedures are provided in Supplementary Methods. Bone-marrow neutrophils from 6–8-week-old male C57BL/6J mice were isolated by discontinuous Percoll density-gradient centrifugation (78%/56%; 600 × g, 30 min), and the neutrophil-enriched fraction was collected. Flow cytometry confirmed that the proportion of Ly6G-positive cells routinely exceeded 90%. Unless otherwise indicated, freshly isolated neutrophils were activated with recombinant murine TNF-α (50 ng/mL, 3 h), and nanoparticles were applied at an Mn_3_O_4_-equivalent concentration of 5 μg/mL for 4 h in all *in vitro* neutrophil treatment experiments.

### 2.7 Cell viability assay

H9c2 cells or fresh neutrophils were seeded in 96-well plates (5 × 10^3^ cells/well). H9c2 cells were allowed to adhere overnight, whereas neutrophils were treated immediately after seeding. Cells were treated for 24 h with PBS or with Mn_3_O_4_ NPs, L@Mn_3_O_4_, or APL@Mn_3_O_4_ at the indicated Mn_3_O_4_-equivalent concentrations. Cell viability was assessed using CCK-8, with absorbance values measured at 450 nm on a microplate reader and normalized to the corresponding untreated controls. NRCM-specific experimental conditions are provided in Supplementary Methods Section S1.3.

### 2.8 Flow cytometry for nanoparticle uptake

Flow-cytometric data were acquired on a NovoCyte 3000 (Agilent) and analyzed with FlowJo v10.1; neutrophils were identified using PE-conjugated anti-mouse Ly6G (Elabscience, E-AB-F1108D). Resting or TNF-α-activated neutrophils were incubated with DiD-labeled L@Mn_3_O_4_ or APL@Mn_3_O_4_ in serum-free medium or medium containing 10% normal, heat-inactivated, or cobra venom factor-treated mouse serum. Unless otherwise stated, normal serum was pooled in-house from C57BL/6J mice; serum and neutrophil donors were not matched. Serum was heat-inactivated at 56 °C for 30 min or treated with cobra venom factor (10 U/mL, 1 h, 37 °C). For antibody-interference experiments, activated neutrophils were preincubated for 30 min at 37 °C with antibodies against mouse CD11b (Abcam, ab237965) or complement component 3 (C3; Abcam, ab225539), each at 0.5 μg/mL. DiD-labeled nanoparticles were then added without washing, and the antibody remained throughout the assay in medium containing 10% pooled normal serum. After incubation, neutrophils were washed three times with cold PBS, and uptake was quantified as the percentage of Ly6G^+^DiD^+^ cells among Ly6G^+^ neutrophils (Fig. S3B). Nanoparticle-associated C3 deposition was assessed by Western blotting as detailed in the Supplementary Methods.

### 2.9 Transwell migration assay

Neutrophil migration was assessed in 12-well Transwell inserts (3.0-μm pores). Activated neutrophils were treated with PBS, Mn_3_O_4_ NPs, L@Mn_3_O_4_, or APL@Mn_3_O_4_. After three washes with cold PBS, 5 × 10^4^ cells in serum-free medium were added per upper chamber. Lower chambers contained serum-free medium without fMLP for the PBS-treated negative control or with fMLP (3 μM) for the PBS-treated fMLP-only positive control and all nanoparticle-treated groups. After 2 h, migrated neutrophils were collected from the lower chambers and counted using an automated cell counter.

### 2.10 Enzyme-linked immunosorbent assay (ELISA)

TNF-α and interleukin-1β (IL-1β) were quantified using mouse ELISA kits (E-EL-M3063 and E-EL-M0037, respectively) according to the manufacturer's instructions; concentrations were calculated from standard curves. Non-activated neutrophils treated with PBS for 4 h served as the resting group. Activated neutrophils were washed three times with PBS to remove residual TNF-α and resuspended in fresh medium before treatment with PBS, Mn_3_O_4_ NPs, L@Mn_3_O_4_, or APL@Mn_3_O_4_ for 4 h. Culture supernatants were clarified by centrifugation (2500 × g, 5 min) and analyzed for both cytokines. In the MI/R model (Section 2.13), heart tissues collected 48 h after reperfusion were homogenized in double-distilled water (10 μL/mg wet tissue) and centrifuged; supernatants were assayed for TNF-α and IL-1β using the same ELISA kits, with concentrations expressed as pg/mL of tissue homogenate.

### 2.11 Neutrophil–cardiomyocyte co-culture model

An indirect neutrophil–cardiomyocyte co-culture was established using 12-well Transwell inserts (0.4-μm pores). H9c2 cells were seeded in the lower chambers (2 × 10^4^ cells/well) and exposed to H_2_O_2_ (150 μM) for 4 h. After removal of the H_2_O_2_-containing medium, cells were washed twice with PBS and cultured in fresh DMEM/RPMI 1640 (1:1, v/v). Activated neutrophils were treated with PBS, Mn_3_O_4_ NPs, L@Mn_3_O_4_, or APL@Mn_3_O_4_, washed three times with cold PBS, resuspended in fresh medium, and added to the upper chambers at the optimized 1:1 neutrophil-to-H9c2 ratio (Supplementary Methods; Fig. S4A). H_2_O_2_-injured H9c2 cells without neutrophils served as the control (CON). After 4 h of co-culture, H9c2 cells were analyzed for intracellular ROS, mitochondrial membrane potential (JC-1 staining), apoptosis, and viability (Calcein-AM/EthD-1 Live/Dead staining). The same design was applied to H_2_O_2_-injured NRCMs (Supplementary Methods).

### 2.12 Intracellular ROS, mitochondrial membrane potential, and apoptosis assays

Freshly isolated, non-activated neutrophils treated with PBS for 4 h served as the resting group, whereas TNF-α-activated neutrophils were treated with PBS, Mn_3_O_4_ NPs, L@Mn_3_O_4_, or APL@Mn_3_O_4_. All groups were subsequently stained with DCFH-DA (10 μM, 20 min, 37 °C) in the dark, washed three times with PBS, and analyzed by flow cytometry. ROS-associated mean fluorescence intensity (MFI) was quantified under identical acquisition settings.

Following the co-culture described in Section 2.11, H9c2 cells were stained with DCFH-DA using the same staining conditions. Fluorescence images were acquired using identical exposure settings across groups, and MFI was quantified. Mitochondrial membrane potential was assessed by JC-1 staining based on the red-to-green fluorescence intensity ratio. H9c2 cells and NRCMs collected after co-culture were stained with an Annexin V-FITC/PI kit. Early and late apoptotic populations were defined as Annexin V^+^/PI^−^ and Annexin V^+^/PI^+^, respectively, and the total apoptotic fraction was calculated as their sum.

### 2.13 Mouse model of myocardial ischemia/reperfusion (MI/R) and treatment protocol

All animal procedures followed institutional guidelines and were approved by the Animal Care and Use Committee of Zhengzhou University (ZZU-LAC20210903(02)). Male C57BL/6J mice (6–8 weeks, 18–22 g) were housed under specific pathogen-free conditions with controlled temperature and humidity, a 12-h light/dark cycle, and ad libitum food and water. Mice were anesthetized with inhaled isoflurane, intubated, and mechanically ventilated; body temperature was maintained with a heating pad. After thoracotomy, the left anterior descending coronary artery (LAD) was ligated for 45 min and released for reperfusion. Postoperative analgesia and monitoring followed the approved protocol. Animals that died intraoperatively, had unsuccessful LAD occlusion or reperfusion, or failed to reach the planned endpoint before sample collection were excluded from analysis.

Unless otherwise indicated, mice received PBS or the indicated nanoparticle formulation at an Mn3O4-equivalent dose of 5 mg kg^−1^ via tail-vein injection 1 h after reperfusion. At 48 h after reperfusion, echocardiography, Evans blue/TTC double staining, myocardial ROS detection, cytokine measurement, and apoptosis assessment were performed. At day 28, echocardiography, Masson's trichrome staining, picrosirius red staining, and CD31/α-SMA dual immunofluorescence were performed.

### 2.14 *In vivo* fluorescence imaging and biodistribution

For *in vivo* tracking, mice were intravenously injected with PBS, DiD-labeled L@Mn_3_O_4_ or APL@Mn_3_O_4_. The same mice underwent longitudinal whole-body fluorescence imaging at 6 h and on days 1, 3, and 7 using an IVIS Spectrum system. At 24 h, an independent cohort was euthanized for *ex vivo* imaging of the heart, liver, spleen, brain, lung, and kidney under identical acquisition settings; signals were quantified as total flux.

In selected experiments, peripheral blood collected 24 h after injection was analyzed by flow cytometry after erythrocyte lysis to determine the proportion and distribution of DiD-positive cells among leukocyte populations. Within CD45^+^ leukocytes, neutrophils were defined as CD11b^+^Ly6G^+^, monocytes as CD11b^+^Ly6G^−^Ly6C^+^ where applicable, and the remaining cells as other leukocytes (Fig. S3A). For each population, the composite DiD-associated signal was calculated as the product of its DiD MFI and its fraction among all DiD-positive peripheral blood cells.

At 24 h, hearts from MI/R mice receiving the same treatments were collected, sectioned, immunostained for Ly6G, and counterstained with DAPI; intrinsic DiD fluorescence was recorded without additional staining. Images were acquired under identical settings, and Ly6G/DiD association was quantified as the percentage of Ly6G^+^DiD^+^ cells among total Ly6G^+^ cells. Field values were averaged per mouse to yield a single biological replicate.

### 2.15 Echocardiography

Cardiac function was assessed by transthoracic echocardiography using a Vevo 2100 system (VisualSonics, Toronto, Canada). Blinded measurements were averaged over three consecutive cardiac cycles per animal. The parameters analyzed included left ventricular ejection fraction (LVEF), left ventricular fractional shortening (LVFS), left ventricular end-systolic volume (LVESV), and left ventricular end-diastolic volume (LVEDV).

### 2.16 TTC staining, histology, and immunofluorescence

At 48 h after reperfusion, the LAD was re-occluded, and 2% Evans blue was retrogradely perfused through the aorta to delineate non-ischemic myocardium. Excised hearts were frozen, transversely sliced, and incubated with 2% TTC (37 °C, 20 min). The area at risk and infarct area were quantified using ImageJ. Infarct size was expressed as infarct area/area at risk (%), and, where indicated, area at risk as a percentage of left ventricular area.

Tissue ROS was assessed in heart cryosections using DHE (10 μM, 30 min). Fluorescence images were acquired under identical exposure settings, and myocardial MFI was quantified using ImageJ. For immunofluorescence, sections were incubated with the indicated primary and corresponding Alexa Fluor-conjugated secondary antibodies. TUNEL-positive nuclei were quantified as a percentage of DAPI-positive nuclei. CD68^+^CD86^+^ and CD68^+^CD206^+^ cells were quantified as percentages of total CD68^+^ cells; for these analyses, DAPI was used only for nuclear counterstaining. No-primary-antibody controls were included (Supplementary Methods; Fig. S5C).

At 28 d after reperfusion, vessel maturation was assessed by CD31/α-SMA dual immunofluorescence, and α-SMA coverage of CD31-positive vessels was quantified using ImageJ. Myocardial fibrosis and collagen deposition were assessed by Masson's trichrome and picrosirius red staining, respectively; the relative collagen type I/III ratio was estimated from the red-to-green birefringence ratio in polarized-light images of picrosirius red-stained sections. For each image-based endpoint, measurements from all analyzed fields, sections, or slices were averaged per mouse, with each mouse treated as one biological replicate in group-level statistical analyses.

### 2.17 Statistical analysis

Data are presented as mean ± standard deviation (SD). *In vitro n* denotes independent experiments and *in vivo n* individual mice; technical replicates were averaged before analysis. Two independent groups were compared using unpaired two-tailed Student's *t*-tests; single-factor comparisons among ≥3 groups used ANOVA. Two-factor data were analyzed by ordinary two-way ANOVA or, when repeated within the same biological unit, by two-way repeated-measures ANOVA; independent time-point cohorts were treated as non-repeated data. Tukey's or Šídák's multiple-comparisons tests were applied as specified in figure legends. Mice were randomized; echocardiographic and image analyses were blinded. Analyses used GraphPad Prism 10; *P* < 0.05 was considered statistically significant.

## 3 Results

### 3.1 Physicochemical characterization of APL@Mn_3_O_4_ and its antioxidant and enzyme-mimetic activities

We first characterized the physicochemical properties of APL@Mn_3_O_4_ and assessed whether liposomal encapsulation and AP functionalization affected the antioxidant activity of the Mn_3_O_4_ core. TEM showed well-dispersed, quasi-spherical Mn_3_O_4_ NPs, whereas APL@Mn_3_O_4_ appeared as a larger liposomal formulation (Fig. 1A and B). DLS showed an increase in mean hydrodynamic diameter from approximately 10 nm for Mn_3_O_4_ NPs to approximately 130 nm for APL@Mn_3_O_4_, accompanied by a shift toward a more negative zeta potential (Fig. 1C, D and Fig. S1A and B). XRD and XPS were then used to examine the crystalline phase and surface chemistry. Powder XRD matched tetragonal hausmannite Mn_3_O_4_ without prominent secondary phases (Fig. S2A). XPS detected Mn, O, and C; Mn 2p peaks at 641.1 and 652.6 eV (ΔE = 11.5 eV), together with a 5.6 eV Mn 3s multiplet splitting, supported mixed Mn(II)/Mn(III) valence, while the O 1s and C 1s spectra were consistent with the expected surface species (Fig. S2B–F). Together, these results confirmed the tetragonal Mn_3_O_4_ phase and its mixed-valence surface chemistry.

**Fig. 1:**
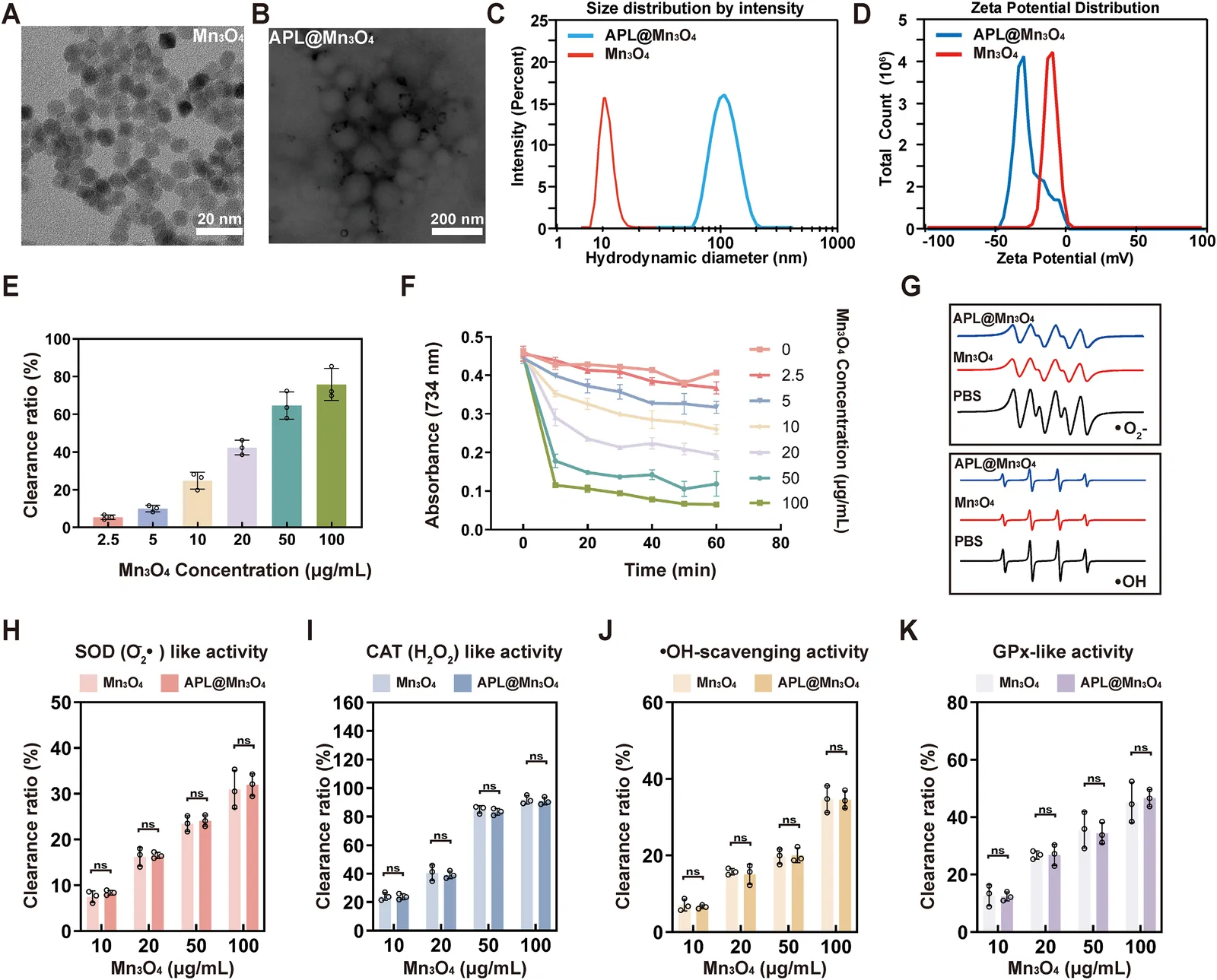
**Physicochemical characterization of APL@Mn_3_O_4_ and evaluation of the antioxidant and enzyme-mimetic activities of the Mn_3_O_4_ core.** (A, B) Representative TEM images of Mn_3_O_4_ NPs (A) and APL@Mn_3_O_4_ (B). Scale bars, 20 nm (A) and 200 nm (B). (C, D) Hydrodynamic size distributions (C) and zeta potentials (D) of Mn_3_O_4_ NPs and APL@Mn_3_O_4_. (E) Concentration-dependent ABTS radical-scavenging activity of APL@Mn_3_O_4_. (F) Time- and concentration-dependent decrease in absorbance at 734 nm with APL@Mn_3_O_4_. (G) ESR analysis of O_2_•^-^ and •OH scavenging by Mn_3_O_4_ NPs and APL@Mn_3_O_4_. (H–K) Concentration-dependent SOD-like (H), CAT-like (I), hydroxyl-radical-scavenging (J), and GPx-like (K) activities of both formulations. Quantitative data are presented as mean ± SD (n = 3). Panel F presents descriptive kinetic profiles. Data in H–K were analyzed using ordinary two-way ANOVA (formulation × concentration) followed by Šídák's multiple-comparisons test comparing formulations at matched concentrations. No significant differences were detected at any matched concentration (all Šídák-adjusted P > 0.05).

We next evaluated surface conjugation and colloidal stability. Relative to L@Mn_3_O_4_, APL@Mn_3_O_4_ showed attenuation of the residual azide band near 2100 cm^−1^ and enhanced IgG-associated amide I and II bands, supporting successful IgG conjugation (Fig. S2G). IgG conjugation and Mn_3_O_4_ encapsulation efficiencies were approximately 86% and 69%, respectively. In PBS at 4 °C and serum at 37 °C, Mn_3_O_4_ NPs and APL@Mn_3_O_4_ showed gradual increases in hydrodynamic diameter over 72 h without abrupt changes, indicating acceptable colloidal stability under the tested conditions (Fig. S2H and I).

Finally, we examined whether formulation affected the antioxidant and enzyme-mimetic activities of the Mn_3_O_4_ core. ABTS assays showed concentration- and time-dependent radical scavenging by APL@Mn_3_O_4_, reaching approximately 75% at 100 μg/mL (Fig. 1E and F). ESR showed that both Mn_3_O_4_ NPs and APL@Mn_3_O_4_ markedly reduced O_2_•^-^ and •OH signals relative to PBS, with similar signal intensities between the two formulations (Fig. 1G). Likewise, both formulations exhibited concentration-dependent SOD-like, CAT-like, hydroxyl radical-scavenging, and GPx-like activities, with no significant between-formulation differences (Fig. 1H–K). Together, these findings support retention of the measured antioxidant and enzyme-mimetic activities of the Mn_3_O_4_ core after formulation.

### 3.2 APL@Mn_3_O_4_ shows preferential uptake by activated neutrophils and attenuates neutrophil oxidative and inflammatory responses *in vitro*

To assess neutrophil-associated delivery of APL@Mn_3_O_4_, we examined neutrophil uptake of the formulations and their effects on neutrophil migration and function *in vitro*. CCK-8 analysis showed minimal effects of Mn_3_O_4_ NPs and APL@Mn_3_O_4_ on neutrophil viability over the tested concentration range (Fig. 2A). L@Mn_3_O_4_ served as an intermediate liposomal control for assessing AP functionalization. Under serum-containing conditions, APL@Mn_3_O_4_ uptake was higher in activated than in resting neutrophils and, among activated neutrophils, higher than L@Mn_3_O_4_ uptake, supporting preferential uptake of APL@Mn_3_O_4_ by activated neutrophils and enhanced uptake following AP functionalization. Removing serum, heat-inactivating serum, or consuming complement with cobra venom factor (CVF) reduced APL@Mn_3_O_4_ uptake; anti-CD11b and anti-C3 treatments likewise reduced uptake by activated neutrophils (Fig. 2B and C). Consistently, immunoblotting showed greater C3 deposition on APL@Mn_3_O_4_ than on L@Mn_3_O_4_ in normal mouse serum, whereas deposition on APL@Mn_3_O_4_ was reduced in heat-inactivated or CVF-treated serum (Fig. S3C). Together, these findings support the involvement of C3-mediated opsonization and complement receptor 3 (CR3; CD11b/CD18) in APL@Mn_3_O_4_ uptake. Residual uptake under serum-free conditions and following interference with the complement pathway suggests that complement-independent mechanisms also contribute.

**Fig. 2:**
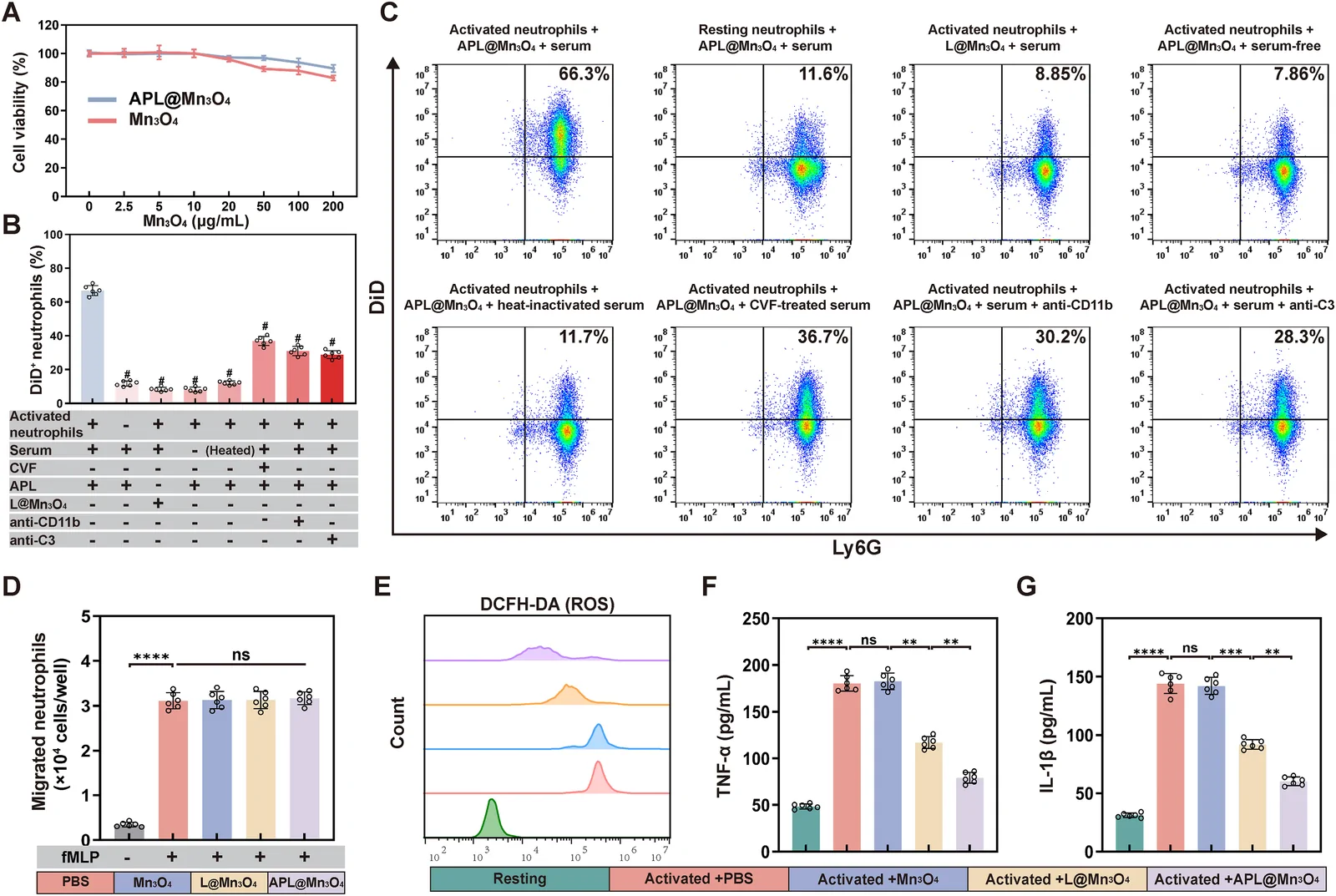
**Neutrophil uptake, migration, and functional regulation by APL@Mn_3_O_4_*in vitro*.** (A) Viability of neutrophils exposed to increasing concentrations of Mn_3_O_4_ NPs or APL@Mn_3_O_4_. (B) Quantification of DiD-labeled nanoparticle uptake by neutrophils under the indicated activation, serum, complement-interference, and CD11b-interference conditions. APL, APL@Mn_3_O_4_; Heated, heat-inactivated serum. (C) Representative flow-cytometric plots showing Ly6G^+^DiD^+^ neutrophils under the indicated conditions. (D) Absolute numbers of neutrophils that migrated during the 2-h Transwell assay; fMLP (3 μM) was present in the lower chamber for the fMLP-only positive-control and all nanoparticle-treated groups. (E) Representative DCFH-DA flow-cytometric histograms showing intracellular ROS levels in neutrophils after the indicated treatments. (F, G) ELISA quantification of TNF-α (F) and IL-1β (G) in neutrophil culture supernatants. For panels E–G, Resting denotes non-activated, PBS-treated neutrophils, whereas all other groups were TNF-α-activated and treated as indicated. Quantitative data are presented as mean ± SD (n = 6). Statistical comparisons in panels B, D, F, and G were performed using one-way ANOVA followed by Tukey's multiple-comparisons test. For panel B, #P < 0.05 versus activated neutrophils treated with APL@Mn_3_O_4_ in normal serum. For panels D, F, and G, *P < 0.05, **P < 0.01, ***P < 0.001, and ****P < 0.0001; ns, not significant. CVF, cobra venom factor.

We next assessed how the nanoparticle formulations affected neutrophil migration and oxidative and inflammatory readouts. An initial 1-h Transwell assay showed no significant differences among the fMLP-treated groups (Fig. S3E). Extending the assay to 2 h yielded the same result: although fMLP (3 μM) markedly increased migration compared with the PBS-treated group without fMLP, none of the nanoparticle formulations significantly altered migration relative to the PBS-treated fMLP group (Fig. 2D). DCFH-DA flow cytometry showed comparable ROS-associated fluorescence in the PBS and Mn_3_O_4_ NP groups, lower fluorescence with L@Mn_3_O_4_, and the lowest fluorescence with APL@Mn_3_O_4_ (Fig. 2E; Fig. S3D and F). Because intracellular ROS can promote pro-inflammatory cytokine expression in neutrophils [22], we next measured cytokine release. TNF-α and IL-1β levels showed the same stepwise pattern, with partial reductions after L@Mn_3_O_4_ and the greatest reductions after APL@Mn_3_O_4_ (Fig. 2F and G). Thus, liposomal encapsulation contributed partially to ROS and cytokine regulation, whereas AP functionalization further enhanced neutrophil uptake and suppression of oxidative and inflammatory responses without measurably impairing migration under the tested conditions.

### 3.3 APL@Mn_3_O_4_-treated neutrophils attenuate H_2_O_2_-induced cardiomyocyte injury in H9c2 cells and NRCMs

To assess how neutrophil pretreatment influenced cardiomyocyte injury, H_2_O_2_-injured H9c2 cells were indirectly co-cultured with activated neutrophils pretreated with PBS, Mn_3_O_4_ NPs, L@Mn_3_O_4_, or APL@Mn_3_O_4_ and washed before co-culture (Fig. 3A). Direct exposure to Mn_3_O_4_ NPs or APL@Mn_3_O_4_ minimally affected H9c2 viability over the tested concentration range (Fig. 3B). DCFH-DA staining showed similarly high ROS levels in the PBS and Mn_3_O_4_ NP groups, lower levels with L@Mn_3_O_4_, and the greatest reduction with APL@Mn_3_O_4_ (Fig. 3C and Fig. S4B). JC-1 staining showed a parallel pattern: marked mitochondrial membrane-potential loss in the PBS and Mn_3_O_4_ NP groups, partial preservation with L@Mn_3_O_4_, and the greatest preservation with APL@Mn_3_O_4_ (Fig. 3D and Fig. S4C). Apoptosis did not differ significantly between the PBS and Mn_3_O_4_ NP groups but decreased with L@Mn_3_O_4_ and further with APL@Mn_3_O_4_ (Fig. 3E and Fig. S4D). Consistently, Live/Dead staining showed a corresponding reduction in cell death (Fig. 3F and Fig. S4E).

**Fig. 3:**
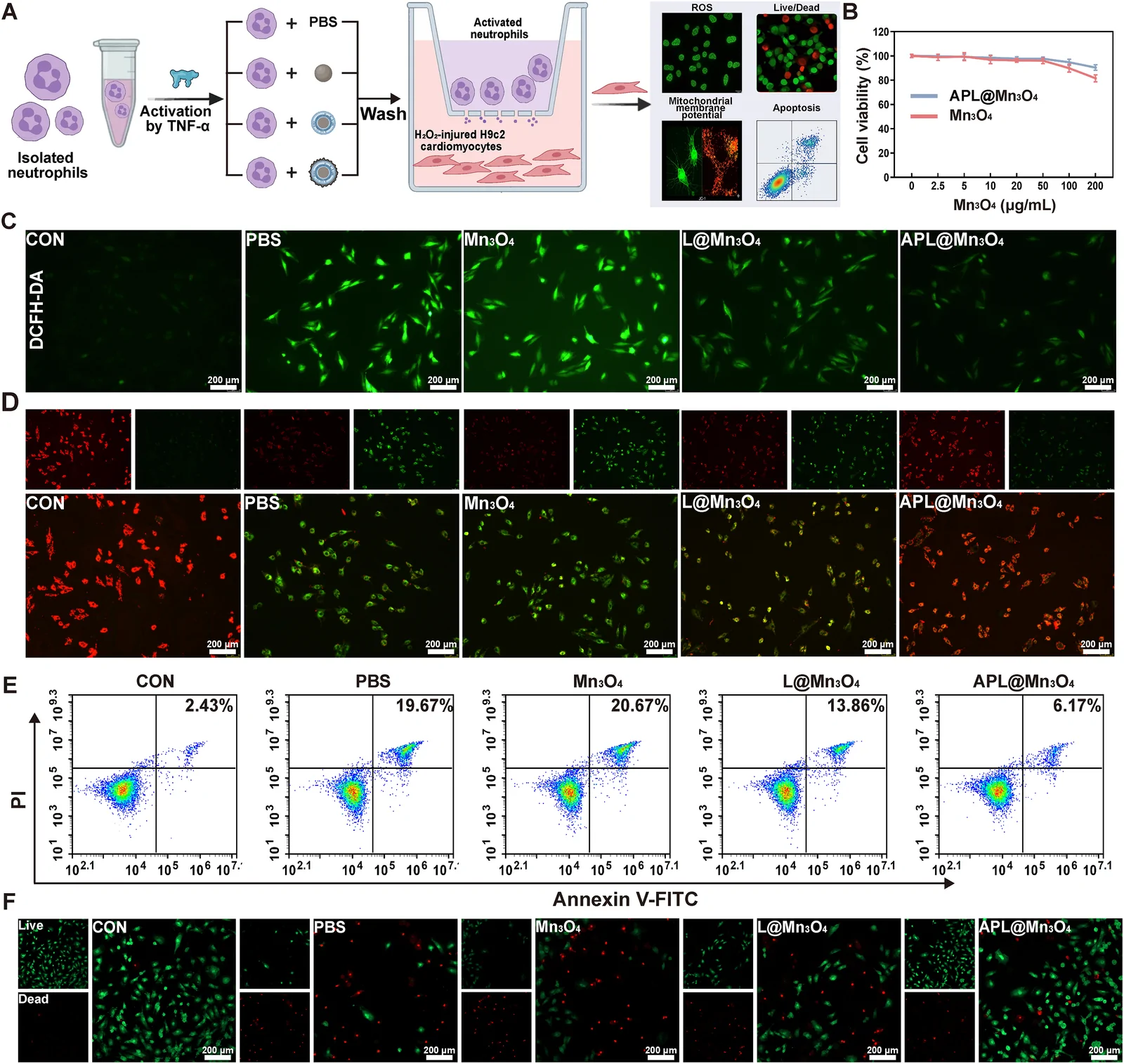
**Effects of differently pretreated neutrophils on H9c2 cells in the co-culture system.** (A) Schematic of the indirect co-culture system used to assess H9c2 injury after co-culture with activated neutrophils pretreated with PBS, Mn_3_O_4_ NPs, L@Mn_3_O_4_, or APL@Mn_3_O_4_. (B) CCK-8 analysis of H9c2 viability after exposure to increasing Mn_3_O_4_-equivalent concentrations of Mn_3_O_4_ NPs or APL@Mn_3_O_4_ (n = 6). (C–F) Representative images or plots of H_2_O_2_-injured H9c2 cells after co-culture under the indicated neutrophil pretreatment conditions: DCFH-DA fluorescence for intracellular ROS (C), JC-1 staining for mitochondrial membrane potential (D; red, aggregates; green, monomers), Annexin V-FITC/PI flow cytometry for apoptosis (E), and Calcein-AM/EthD-1 Live/Dead staining (F). CON denotes H_2_O_2_-injured H9c2 cells cultured without neutrophils. Scale bars, 200 μm (C, D, and F). Data in B are mean ± SD (n = 6) and were analyzed by two-way ANOVA (formulation × concentration) with Šídák's multiple-comparisons test. (For interpretation of the references to colour in this figure legend, the reader is referred to the Web version of this article.)

To determine whether these findings extended to primary cardiomyocytes, we next evaluated isolated NRCMs, whose cardiomyocyte identity was verified by sarcomeric α-actinin staining (Fig. S4F). NRCM viability remained above 80% after direct 24-h exposure to Mn_3_O_4_ NPs or APL@Mn_3_O_4_ at concentrations up to 100 μg/mL, indicating acceptable cytocompatibility under the tested conditions (Fig. S4G). We then assessed apoptosis in the NRCM co-culture model. Compared with H_2_O_2_-injured NRCMs cultured without neutrophils, co-culture with PBS-pretreated activated neutrophils increased apoptosis. Apoptosis did not differ significantly between the PBS and Mn_3_O_4_ NP pretreatment groups, was lower with L@Mn_3_O_4_ than with Mn_3_O_4_ NPs, and was further reduced with APL@Mn_3_O_4_ relative to L@Mn_3_O_4_ (Fig. S4H and I). Together with the preceding evidence of attenuated neutrophil oxidative and inflammatory responses, these concordant findings in H9c2 cells and NRCMs support cardiomyocyte protection associated with liposomal encapsulation and further enhanced by AP functionalization.

### 3.4 APL@Mn_3_O_4_ shows preferential association with circulating neutrophils and increased heart-associated fluorescence in MI/R mice

To evaluate neutrophil-associated delivery *in vivo*, we compared the biodistribution and circulating-cell association of DiD-labeled L@Mn_3_O_4_ and APL@Mn_3_O_4_ following intravenous administration in MI/R mice. At 24 h, DiD-associated APL@Mn_3_O_4_ signals were predominantly detected in CD45^+^ leukocytes, whereas L@Mn_3_O_4_ showed a larger CD45^−^ fraction (Fig. 4A and B). Neutrophils accounted for 70.54% of DiD^+^ leukocytes in the APL@Mn_3_O_4_ group versus 38.96% in the L@Mn_3_O_4_ group. Quantification as MFI × fraction of cells likewise showed a higher neutrophil-associated signal for APL@Mn_3_O_4_, whereas L@Mn_3_O_4_ produced relatively higher signals in CD45^−^ cells, monocytes, and other leukocytes (Fig. 4C), supporting preferential circulating-neutrophil association after AP functionalization.

**Fig. 4:**
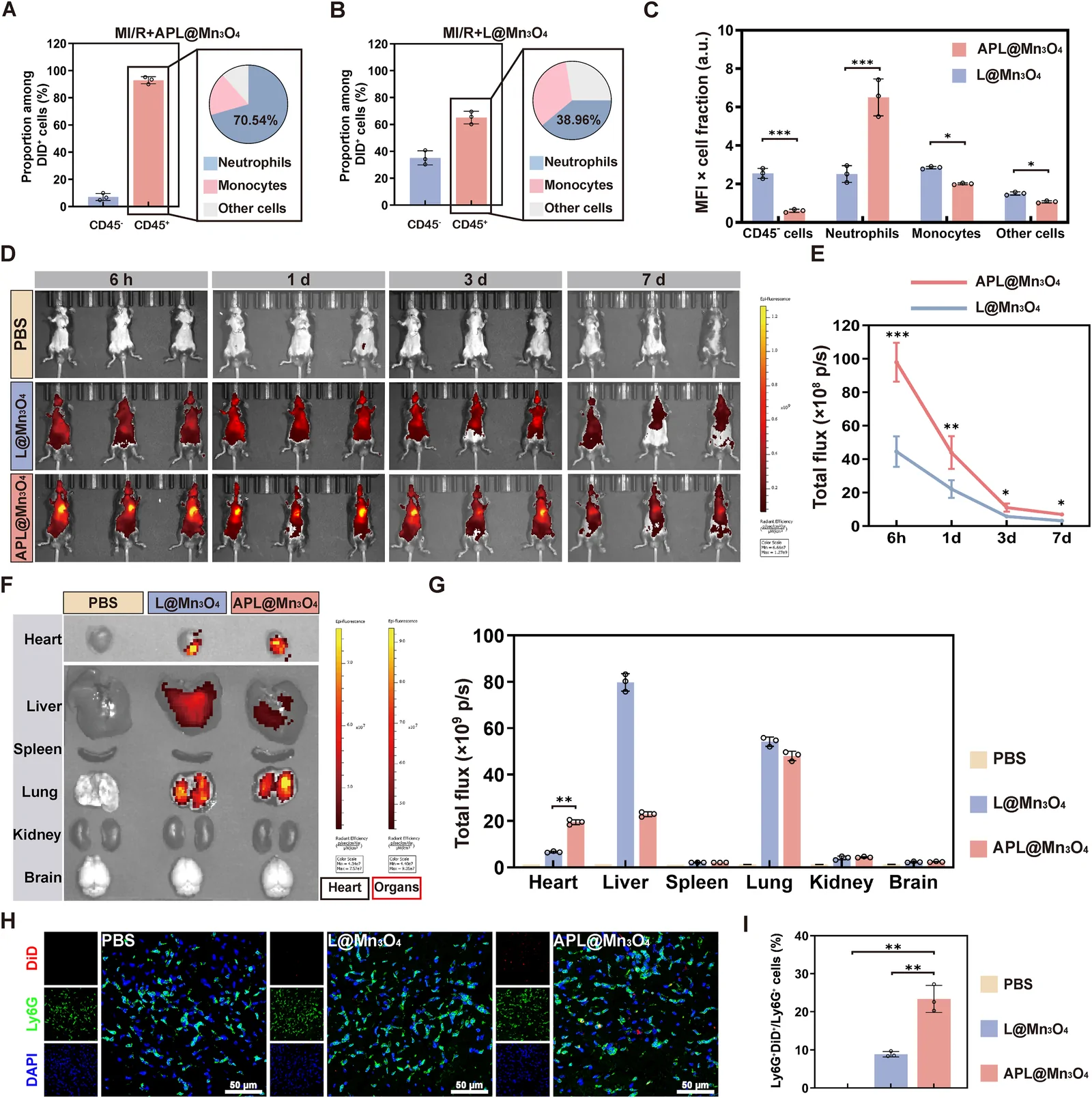
***In vivo* biodistribution and circulating-cell association of L@Mn_3_O_4_ and APL@Mn_3_O_4_.** MI/R mice received PBS, DiD-labeled L@Mn_3_O_4_, or DiD-labeled APL@Mn_3_O_4_ intravenously. Panels A–C and F–I were assessed at 24 h. (A, B) Flow-cytometric analysis of DiD-positive peripheral blood cells. (C) Nanoparticle-associated signals across circulating cell populations, calculated as MFI × cell fraction. (D, E) Representative whole-body fluorescence images at 6 h and days 1, 3, and 7 (D) and corresponding total-flux quantification (E). (F, G) *Ex vivo* fluorescence images of the heart and major organs (F) and corresponding total-flux quantification (G). (H, I) Representative myocardial fluorescence images showing Ly6G (green), DiD (red), DAPI (blue), and merged signals (H), with Ly6G^+^DiD^+^ cells quantified as a percentage of Ly6G^+^ cells (I). Scale bar, 50 μm. Quantitative data are mean ± SD (n = 3 mice per group). Panels C, E, and G were analyzed by two-way repeated-measures ANOVA with Šídák's multiple-comparisons test, and panel I by one-way ANOVA with Tukey's multiple-comparisons test. In E, symbols above APL@Mn_3_O_4_ indicate comparisons with L@Mn_3_O_4_ at matched time points; only the prespecified heart comparison is shown in G. ns, not significant; *P < 0.05, **P < 0.01, ***P < 0.001, and ****P < 0.0001. (For interpretation of the references to colour in this figure legend, the reader is referred to the Web version of this article.)

Whole-body imaging of the DiD-labeled formulations showed stronger APL@Mn_3_O_4_ fluorescence, particularly at 6 h, followed by progressive attenuation through day 7 (Fig. 4D and E). Ex vivo imaging at 24 h showed systemic distribution with prominent liver and lung signals for both formulations, but higher heart-associated fluorescence with APL@Mn_3_O_4_ (Fig. 4F and G). Myocardial sections also showed greater Ly6G/DiD colocalization and a higher proportion of Ly6G^+^DiD^+^ cells among total Ly6G^+^ cells with APL@Mn_3_O_4_ than with L@Mn_3_O_4_, whereas the PBS group showed negligible colocalization (Fig. 4H and I). Collectively, these findings demonstrate that AP functionalization enhances neutrophil-associated delivery and cardiac accumulation of DiD-labeled APL@Mn_3_O_4_ following reperfusion.

### 3.5 APL@Mn_3_O_4_ reduces acute myocardial injury and oxidative and inflammatory readouts after reperfusion

To determine whether the stepwise advantages observed *in vitro* translated into early myocardial protection after reperfusion, we assessed acute injury following APL@Mn_3_O_4_ treatment *in vivo*. At 48 h, echocardiography showed improved cardiac function with APL@Mn_3_O_4_, reflected by increased LVEF and LVFS and reduced LVESV and LVEDV; L@Mn_3_O_4_ produced a partial but less pronounced improvement relative to Mn_3_O_4_ NPs and PBS (Fig. 5A–E). Consistently, no statistically significant differences in area at risk (AAR) relative to left ventricular area were detected among groups, but infarct size normalized to the AAR was reduced with L@Mn_3_O_4_ and further reduced with APL@Mn_3_O_4_, indicating partial protection from liposomal encapsulation and additional benefit from AP functionalization (Fig. 5F and Fig. S5A).

**Fig. 5:**
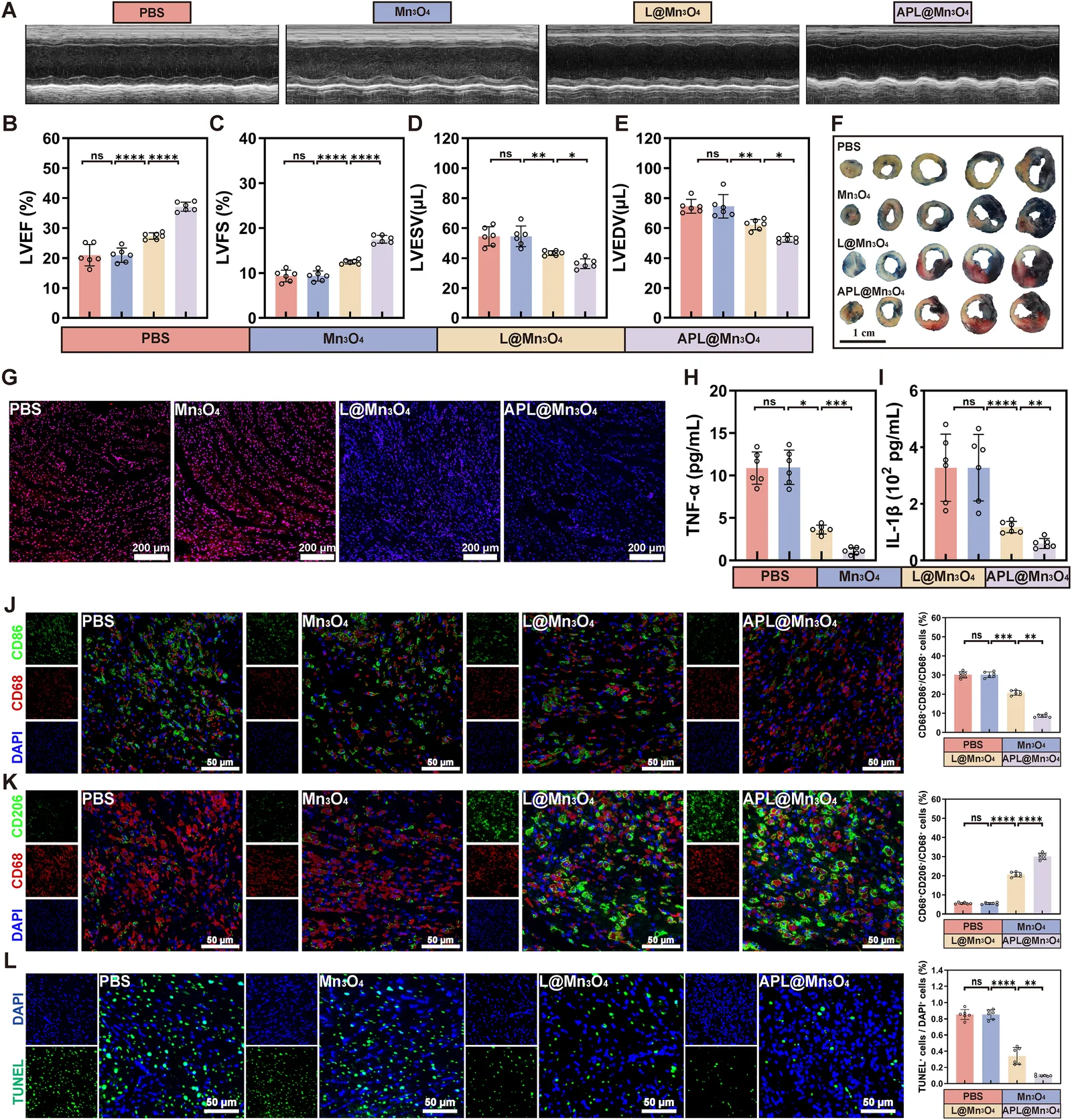
**Acute myocardial injury after different nanoparticle treatments *in vivo*.** (A–E) Representative M-mode echocardiograms at 48 h after reperfusion in mice treated with PBS, Mn_3_O_4_ NPs, L@Mn_3_O_4_, or APL@Mn_3_O_4_ (A), with quantification of LVEF (B), LVFS (C), LVESV (D), and LVEDV (E). (F) Representative Evans blue/TTC-stained heart sections showing non-ischemic (blue), AAR (red), and infarct (white) areas. Scale bar, 1 cm. (G) Representative DHE fluorescence images of myocardial ROS with DAPI counterstaining. Scale bar, 200 μm. (H, I) Myocardial TNF-α (H) and IL-1β (I) quantified by ELISA. (J, K) Representative high-magnification immunofluorescence images and quantification of CD68^+^CD86^+^ (J) and CD68^+^CD206^+^ (K), expressed as percentages of total CD68^+^ cells. CD68 is shown in red, CD86 or CD206 in green, and DAPI in blue. Scale bars, 50 μm. (L) Representative myocardial TUNEL staining and quantification of TUNEL-positive nuclei as a percentage of total DAPI-positive nuclei. Scale bar, 50 μm. Data are presented as mean ± SD (n = 6) and were analyzed by one-way ANOVA followed by Tukey's multiple-comparisons test. ns, not significant; *P < 0.05, **P < 0.01, ***P < 0.001, and ****P < 0.0001. (For interpretation of the references to colour in this figure legend, the reader is referred to the Web version of this article.)

We next examined tissue-level oxidative and inflammatory injury. DHE staining showed reduced myocardial ROS after L@Mn_3_O_4_ treatment and a further reduction after APL@Mn_3_O_4_ treatment (Fig. 5G and Fig. S5B). Because excessive ROS can promote inflammatory activation, we assessed cytokines and inflammation-associated markers in the infarcted myocardium. ELISA showed a similar stepwise pattern: L@Mn_3_O_4_ partially reduced myocardial TNF-α and IL-1β, whereas APL@Mn_3_O_4_ produced the greatest reduction (Fig. 5H and I). To assess macrophage phenotype-associated changes, myocardial sections were co-stained for CD68 and CD86 or for CD68 and CD206. Compared with PBS, APL@Mn_3_O_4_ was associated with a lower proportion of CD68^+^CD86^+^ cells and a higher proportion of CD68^+^CD206^+^ cells among total CD68^+^ cells (Fig. 5J and K), indicating an altered macrophage marker profile in the local myocardial microenvironment under the tested conditions. Because oxidative and inflammatory stress can converge on apoptotic injury, we next assessed myocardial apoptosis. TUNEL staining showed reduced apoptotic injury with L@Mn_3_O_4_ and the greatest reduction with APL@Mn_3_O_4_ (Fig. 5L). Together, these results indicate that liposomal encapsulation alone partially contributed to early myocardial protection, whereas AP functionalization further strengthened reductions in oxidative stress, inflammatory readouts, and apoptotic injury *in vivo*. In a parallel preliminary biosafety assessment of the MI/R treatment cohorts, serum liver- and kidney-related biochemical indices showed no obvious abnormalities among the four groups at 48 h, and hematoxylin and eosin (H&E) staining of the liver, spleen, lung, and kidney revealed no evident treatment-related histopathological changes (Fig. S5D and E).

### 3.6 APL@Mn_3_O_4_ improves post-reperfusion functional recovery and remodeling-associated readouts

Because early myocardial protection may influence subsequent remodeling, we assessed post-reperfusion cardiac function and structural changes at day 28. Echocardiography showed improved function with APL@Mn_3_O_4_, reflected by increased LVEF and LVFS and reduced LVESV and LVEDV; L@Mn_3_O_4_ produced a partial but less pronounced improvement relative to PBS and bare Mn_3_O_4_ NPs (Fig. 6A–E). We next determined whether functional recovery was accompanied by histological remodeling. Masson's trichrome staining showed reduced myocardial fibrosis with L@Mn_3_O_4_ and a greater reduction with APL@Mn_3_O_4_ (Fig. 6F–H). Picrosirius red staining under polarized light similarly showed a lower collagen type I/III ratio with L@Mn_3_O_4_ and a further reduction with APL@Mn_3_O_4_ (Fig. 6G–I).

**Fig. 6:**
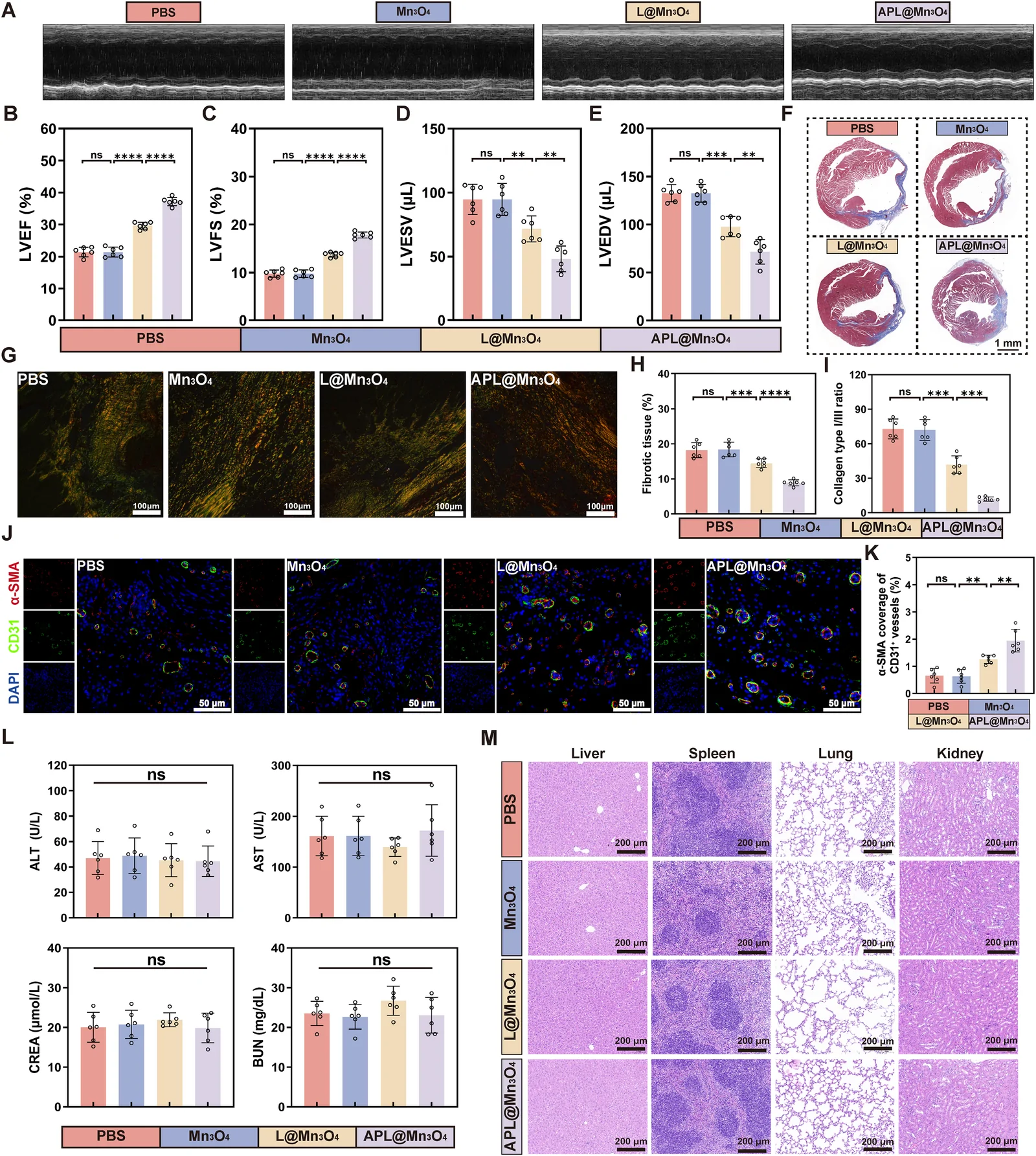
**Day-28 cardiac remodeling and vessel-maturity-associated readouts after nanoparticle treatment.** (A) Representative M-mode echocardiograms after reperfusion in mice treated with PBS, Mn_3_O_4_ NPs, L@Mn_3_O_4_, or APL@Mn_3_O_4_. (B–E) LVEF (B), LVFS (C), LVESV (D), and LVEDV (E). (F, H) Representative Masson's trichrome-stained sections (F; scale bar, 1 mm) and quantification of fibrotic area (H). (G, I) Representative picrosirius red-stained sections under polarized light (G; scale bar, 100 μm) and quantification of the collagen type I/III ratio (I). (J, K) Representative myocardial immunofluorescence for CD31 (green), α-SMA (red), and DAPI (blue) (J; scale bar, 50 μm) and quantification of α-SMA coverage of CD31-positive vessels (K). (L) Serum liver- and kidney-function indices. (M) Representative H&E-stained sections of major non-cardiac organs (scale bar, 200 μm). Data are mean ± SD (n = 6). One-way ANOVA with Tukey's multiple-comparisons test. ns, not significant; *P < 0.05, **P < 0.01, ***P < 0.001, ****P < 0.0001. (For interpretation of the references to colour in this figure legend, the reader is referred to the Web version of this article.)

We next assessed structural features of vessel maturation using dual CD31/α-SMA immunofluorescence. α-SMA coverage of CD31-positive vessels did not differ between the PBS and Mn_3_O_4_ NP groups but was progressively higher in the L@Mn_3_O_4_ and APL@Mn_3_O_4_ groups (Fig. 6J and K). These findings support enhanced vessel maturation at the structural level. Overall, the day-28 data indicate that liposomal encapsulation partially improved cardiac function and attenuated adverse remodeling, with further benefits from AP functionalization. At the same time point, serum markers of liver and kidney function showed no treatment-related changes, and H&E-stained sections of the liver, spleen, lung, and kidney showed no evident abnormalities (Fig. 6L and M).

### 3.7 Preliminary systemic tolerability and temporal tissue Mn disposition

Independent cohorts of healthy mice were evaluated at 48 h and day 28 after a single intravenous administration of APL@Mn_3_O_4_ or PBS. No significant between-group differences were observed in red blood cell (RBC), white blood cell (WBC), or platelet (PLT) counts or in serum TNF-α, IL-1β, or interleukin-6 (IL-6) levels at either time point (Fig. S6A–F). In separate cohorts of MI/R mice, ICP-MS analysis showed that total tissue Mn concentrations after APL@Mn_3_O_4_ administration were highest at 24 h and declined thereafter. At day 7, Mn levels remained higher than those in the corresponding PBS groups in most organs, whereas no significant between-group differences were detected in any examined organ at day 28 (Fig. S6G–L). Together with the absence of overt abnormalities in serum indices of liver and kidney function or in major non-cardiac organ histology at 48 h and day 28 (Figs. S5D–E and 6L–M), these findings provide preliminary support for the systemic tolerability of a single APL@Mn_3_O_4_ dose and argue against progressive organ Mn accumulation over the 28-day observation period.

## 4 Discussion

Our findings show that APL@Mn_3_O_4_ integrates Mn_3_O_4_-mediated multienzyme-like antioxidant activity with an agglutinated-protein liposome architecture that facilitates association with activated neutrophils under inflammatory conditions. This integrated platform effectively attenuated oxidative and inflammatory myocardial injury, protected cardiomyocytes, improved cardiac function and long-term ventricular remodeling after MI/R, and exhibited favorable systemic tolerability. MIRI is driven not simply by ROS overload but by a reperfusion-triggered cascade in which oxidative stress and inflammatory activation reinforce one another [2], [3], [23]. Neutrophils act as early upstream effectors in this cascade, aggravating tissue injury through ROS, proteases, and pro-inflammatory mediators [4], [5], [24]. However, strategies that suppress neutrophil recruitment or broadly dampen inflammatory amplification may not selectively restrain these pathological effector functions and may also interfere with neutrophil-dependent inflammation resolution and tissue repair [16], [17]. Together, these considerations support interventions that selectively restrain pathological neutrophil output through redox regulation.

Although several nanozyme systems have shown therapeutic potential in MIRI [25], [26], [27], [28], an appropriate catalytic core should combine broad-spectrum ROS regulation, low pro-oxidant liability, and biocompatibility [29], [30]. Some reported cardiac nanozyme platforms, however, primarily rely on SOD-like or SOD/CAT-like activities [14], [25], [31], which may not fully address the complex and progressively amplified oxidative injury in MIRI. Moreover, some reported nanozyme systems involve peroxidase- or oxidase-like reaction pathways that may introduce pro-oxidant risk, whereas others rely on noble-metal or non-physiological cores, including Pt or Pt-doped ceria [26], [27], whose long-term safety warrants careful evaluation [32], [33], [34]. We selected Mn_3_O_4_ as the catalytic core for two main reasons. First, Mn_3_O_4_ exhibits SOD-, CAT-, and GPx-like activities [15], [35], providing a catalytic profile suited to the complex oxidative stress landscape of MIRI. Second, manganese is an essential trace element involved in normal physiology, providing a physiologically relevant elemental basis that distinguishes Mn_3_O_4_ from noble-metal and other non-physiological catalytic cores [36]. Consistent with this design rationale, the broad-spectrum antioxidant activities of the Mn_3_O_4_ core were retained after liposomal encapsulation and AP functionalization. In parallel, the absence of evident treatment-related abnormalities across the evaluated hematological, inflammatory, biochemical, and histopathological endpoints provides preliminary support for systemic tolerability after a single dose, and the time-dependent decline in tissue Mn concentrations argues against progressive organ Mn accumulation over the 28-day observation period.

Neutrophils are rapidly recruited to the reperfused myocardium, where they amplify secondary oxidative and inflammatory injury through the release of ROS, proteases, and pro-inflammatory mediators [24]. Neutrophil recruitment into injured myocardium is regulated by mechanisms including CCR2^+^ macrophage-dependent TLR9/MyD88/CXCL5 signaling [37] and CXCR2-dependent recruitment dynamics [38]. Accordingly, therapeutic strategies have been developed to limit neutrophil adhesion or infiltration, including endothelial-targeted siVCAM-1/dexamethasone nanocomplexes [39], anti-CD11b blockade [40], and CXCR2 antagonism with navarixin [41]. However, neutrophils are not solely injurious effector cells in the infarcted myocardium; they also participate in inflammation resolution and post-infarction repair. Neutrophil depletion after myocardial infarction worsens cardiac function, increases fibrosis, and impairs reparative macrophage polarization [42], whereas enhanced efferocytosis of apoptotic neutrophil-derived bodies promotes inflammation resolution and infarct repair [43]. Neutrophil-associated pro-resolving pathways, including Annexin A1-dependent macrophage programming, have also been linked to vascular endothelial growth factor A (VEGF-A) production and post-infarction neovascularization, whereas impaired resolution signaling increases the risk of cardiac hemorrhage and rupture [44], [45]. Broad inhibition of neutrophil recruitment or function may therefore provide acute benefit while compromising subsequent healing.

These findings favor selective restraint of pathological neutrophil outputs over broad suppression. Accordingly, APL@Mn_3_O_4_ was designed not to eliminate neutrophils or block their recruitment, but rather to preferentially engage activated neutrophils under inflammatory conditions and attenuate their pathological oxidative and inflammatory responses through Mn_3_O_4_-mediated catalytic redox regulation. The reductions in neutrophil-associated ROS and inflammatory cytokine-related readouts, together with the absence of measurable impairment in migration under the tested conditions, suggest that APL@Mn_3_O_4_ attenuates pathological neutrophil-associated oxidative and inflammatory activity without measurably impairing the migratory response assessed here. Mechanistically, APL@Mn_3_O_4_ associated more strongly with activated neutrophils than L@Mn_3_O_4_, particularly in normal serum. Greater nanoparticle-associated C3 deposition for APL@Mn_3_O_4_ than for L@Mn_3_O_4_ in normal serum, together with reduced association after serum heat inactivation, CVF treatment, anti-C3 treatment, or CD11b blockade, collectively supports C3-associated opsonization and involvement of CD11b-containing CR3. Because these interventions attenuated rather than abolished the association, with residual association after CVF treatment remaining above serum-free levels, the tested complement- and CR3-associated mechanisms appear contributory rather than exclusive. This residual association may reflect incomplete complement consumption or additional complement-independent interactions. *In vivo*, DiD-labeled APL@Mn_3_O_4_ showed greater association with circulating neutrophils than L@Mn_3_O_4_, accompanied by stronger heart-associated fluorescence and increased myocardial Ly6G/DiD colocalization. Although altered circulation behavior or passive accumulation in injured myocardium may also contribute to the cardiac signal, these findings support a neutrophil-associated component of the enhanced myocardial delivery of DiD-labeled APL@Mn_3_O_4_.

APL@Mn_3_O_4_-mediated attenuation of neutrophil-associated oxidative and inflammatory activity was accompanied by downstream myocardial protection. Because cardiomyocytes are highly vulnerable to oxidative stress-induced mitochondrial dysfunction and apoptosis [7], [8], [24], limiting neutrophil-associated oxidative and inflammatory stress may reduce secondary injury. In the indirect co-culture system, APL@Mn_3_O_4_-pretreated neutrophils were associated with reduced cardiomyocyte ROS, preserved mitochondrial membrane potential, and less apoptosis, paralleling reduced acute myocardial injury and improved cardiac function *in vivo*. *In vivo*, lower inflammatory cytokine levels, together with a lower proportion of CD68^+^CD86^+^ cells and a higher proportion of CD68^+^CD206^+^ cells within the total CD68^+^ population, were consistent with an attenuated inflammatory milieu and an altered macrophage marker profile. The altered macrophage marker profile may reflect reduced exposure to neutrophil-derived inflammatory mediators, direct effects of Mn_3_O_4_-containing formulations on macrophages, or both. The early protective effects were also associated with improved late functional recovery and reduced adverse remodeling, including fibrosis. The stepwise increase in α-SMA coverage of CD31-positive vessels was consistent with vessel-maturity-associated remodeling, although functional perfusion was not assessed. Together, these findings link attenuation of pathological neutrophil responses with cardiomyocyte protection, reduced acute myocardial injury, and more favorable post-reperfusion remodeling.

Several limitations should be acknowledged. First, although the anti-apoptotic effect observed in H9c2 cells was reproduced in NRCMs, these models may not fully recapitulate adult or human cardiomyocyte responses, and nanoparticle carryover or direct cardiomyocyte effects cannot be completely excluded in the indirect co-culture system. Second, because migration was the principal neutrophil functional readout, preservation of broader neutrophil functions or host defense was not established. Third, although DiD imaging and ICP-MS provided complementary information on DiD-associated distribution and total tissue Mn, they do not directly track intact APL@Mn_3_O_4_; longer-term and repeated-dose studies would further clarify its degradation, clearance, and safety. Within these limits, the findings support APL@Mn_3_O_4_ as a biologically informed nanozyme platform for alleviating MIRI by attenuating pathological neutrophil-associated oxidative and inflammatory activity.

## 5 Conclusion

Overall, this study identifies APL@Mn_3_O_4_ as an agglutinated-protein liposome nanozyme platform that combines preserved catalytic antioxidant activity with complement-associated neutrophil tropism under inflammatory conditions. APL@Mn_3_O_4_ attenuated pathological neutrophil-associated oxidative and inflammatory output, reduced acute myocardial injury, and was associated with improved post-reperfusion functional recovery and remodeling. These findings support neutrophil-tropic redox regulation as a potential strategy for limiting MIRI.

## CRediT authorship contribution statement

**Fu-hang Wang:** Writing – original draft, Visualization, Methodology, Investigation, Formal analysis, Data curation, Conceptualization. **Yuan-song Zhuang:** Methodology, Investigation. **Jun-jie Ma:** Validation, Data curation. **Yu-hang Li:** Methodology, Investigation, Formal analysis. **Quan-xu An:** Writing – review & editing, Resources, Methodology. **De-liang Shen:** Writing – review & editing, Supervision, Project administration, Funding acquisition, Conceptualization.

## Ethics statement

All animal procedures were conducted in accordance with institutional guidelines for the care and use of laboratory animals and were approved by the Animal Care and Use Committee of Zhengzhou University under Approval No. ZZU-LAC20210903(02).

## Funding

This work was supported by the Henan Provincial Key Research and Development Program (Grant No. 241111311600 to De-liang Shen), administered by the Science and Technology Department of Henan Province.

## Declaration of competing interests

The authors declare that they have no known competing financial interests or personal relationships that could have appeared to influence the work reported in this paper.

## Data Availability

The data supporting the findings of this study are available within the article and its Supplementary Material. Additional data are available from the corresponding author upon reasonable request.

## References

[1] González-Montero J., Brito R., Gajardo A.I., Rodrigo R. (2018). Myocardial reperfusion injury and oxidative stress: therapeutic opportunities. World J. Cardiol..

[2] Xiang M., Lu Y., Xin L., Gao J., Shang C., Jiang Z. (2021). Role of oxidative stress in reperfusion following myocardial ischemia injury. Oxid. Med. Cell. Longev..

[3] Frangogiannis N.G. (2014). The inflammatory response in myocardial injury, repair, and remodelling. Nat. Rev. Cardiol..

[4] Silvestre-Roig C., Braster Q., Ortega-Gomez A., Soehnlein O. (2020). Neutrophils as regulators of cardiovascular inflammation. Nat. Rev. Cardiol..

[5] Ma Y., Yabluchanskiy A., Lindsey M.L. (2021). Role of neutrophils in cardiac injury and repair following myocardial infarction. Cells.

[6] Lian Y., Lai X., Wu C., Wang L., Shang J., Zhang H., Jia S., Xing W., Liu H. (2025). The roles of neutrophils in cardiovascular diseases. Front. Cardiovasc. Med..

[7] Ong S.B., Gustafsson Å.B. (2012). New roles for mitochondria in cell death in the reperfused myocardium. Cardiovasc. Res..

[8] Dhalla N.S., Ostadal P., Tappia P.S. (2025). Involvement of oxidative stress and antioxidants in modification of cardiac dysfunction due to ischemia-reperfusion injury. Antioxidants.

[9] Chen H., Tang Y., Ren P., Wu W. (2025). The unmet promise: a critical review of antioxidant strategies in myocardial ischemia-reperfusion injury and the path towards precision medicine. Front. Pharmacol..

[10] Tong C., Zhou B. (2025). Cardioprotective strategies in myocardial ischemia-reperfusion injury: implications for improving clinical translation. J. Mol. Cell. Cardiol..

[11] Wang Q., Zuurbier C.J., Huhn R., Torregroza C., Hollmann M.W., Preckel B. (2023). Pharmacological cardioprotection against ischemia reperfusion injury—the search for a clinical effective therapy. Cells.

[12] Thao N.T.M., Do M.H., Le M.T., Nguyen D.H. (2023). Antioxidant nanozymes: mechanisms, activity manipulation and biomedical applications. Micromachines.

[13] Ren X., Chen D.X., Wang Y., Li H., Li X., Bian Y. (2022). Nanozymes—Recent development and biomedical applications. J. Nanobiotechnol..

[14] Long M., Wang L., Kang L., Liu D., Long T., Ding H., Duan Y., He H., Xu B., Gu N. (2025). Prussian blue nanozyme featuring enhanced superoxide dismutase-like activity for myocardial ischemia reperfusion injury treatment. ACS Nano.

[15] Singh N., Savanur M.A., Srivastava S., D'Silva P., Mugesh G. (2017). A redox modulatory Mn3O4 nanozyme with multi-enzyme activity provides efficient cytoprotection to human cells in a Parkinson's disease model. Angew. Chem. Int. Ed..

[16] Jiang K., Hwa J., Xiang Y. (2024). Novel strategies for targeting neutrophil against myocardial infarction. Pharmacol. Res..

[17] Köhler D., Leiss V., Beichert L., Killinger S., Grothe D., Kushwaha R., Schröter A., Roslan A., Eggstein C., Focken J., Granja T., Devanathan V., Schittek B., Lukowski R., Weigelin B., Rosenberger P., Nürnberg B., Beer-Hammer S. (2024). Targeting Gαi2 in neutrophils protects from myocardial ischemia reperfusion injury. Basic Res. Cardiol..

[18] Myerson J.W., Patel P.N., Rubey K.M., Zamora M.E., Zaleski M.H., Habibi N., Walsh L.R., Lee Y.-W., Luther D.C., Ferguson L.T., Marcos-Contreras O.A., Glassman P.M., Mazaleuskaya L.L., Johnston I., Hood E.D., Shuvaeva T., Wu J., Zhang H.-Y., Gregory J.V., Kiseleva R.Y., Nong J., Grosser T., Greineder C.F., Mitragotri S., Worthen G.S., Rotello V.M., Lahann J., Muzykantov V.R., Brenner J.S. (2022). Supramolecular arrangement of protein in nanoparticle structures predicts nanoparticle tropism for neutrophils in acute lung inflammation. Nat. Nanotechnol..

[19] Chen K., Zheng C., Lv Y., Zhao P., Lin T., Xu Y. (2024). Complement opsonized protein corona activated by precoated immunoglobulin enables neutrophil-hitchhiking for rapid and enhanced drug delivery for acute liver failure recovery. Nano Today.

[20] Inturi S., Wang G., Chen F., Banda N.K., Holers V.M., Wu L. (2015). Modulatory role of surface coating of superparamagnetic iron oxide nanoworms in complement opsonization and leukocyte uptake. ACS Nano.

[21] Che J., Najer A., Blakney A.K., McKay P.F., Bellahcene M., Winter C.W. (2020). Neutrophils enable local and non-invasive liposome delivery to inflamed skeletal muscle and ischemic heart. Adv. Mater..

[22] Mitra S., Abraham E. (2006). Participation of superoxide in neutrophil activation and cytokine production. Biochim. Biophys. Acta Mol. Basis Dis..

[23] Jordan J.E., Zhao Z.-Q., Vinten-Johansen J. (1999). The role of neutrophils in myocardial ischemia-reperfusion injury. Cardiovasc. Res..

[24] El Kazzi M., Rayner B.S., Chami B., Dennis J.M., Thomas S.R., Witting P.K. (2020). Neutrophil-mediated cardiac damage after acute myocardial infarction: significance of defining a new target cell type for developing cardioprotective drugs. Antioxid. Redox Signal..

[25] Xiang K., Wu H., Liu Y., Wang S., Li X., Yang B., Zhang Y., Ma L., Lu G., He L., Ni Q., Zhang L. (2023). MOF-derived bimetallic nanozyme to catalyze ROS scavenging for protection of myocardial injury. Theranostics.

[26] Ye T., Chen C., Wang D., Huang C., Yan Z., Chen Y., Jin X., Wang X., Ding X., Shen C. (2024). Protective effects of Pt-N-C single-atom nanozymes against myocardial ischemia-reperfusion injury. Nat. Commun..

[27] Pu A., Sim W.S., Ji Y., Kurian A.G., Lee J.H., Bui T.V.A., Lai Y., Hwangbo H., Sun H., Kim H.W., Park H.J., Ban K. (2025). Single-atom Pt-doped ceria nanozymes mitigate myocardial ischemia reperfusion injury via cardiomyocyte-targeted uptake and suppression of reactive oxygen species. Bioact. Mater..

[28] Han D., Wang F., Shen D. (2024). Nanomedicines as guardians of the heart: unleashing the power of antioxidants to alleviate myocardial ischemic injury. Theranostics.

[29] Tagaras N., Song H., Sahar S., Tong W., Mao Z., Buerki-Thurnherr T. (2024). Safety landscape of therapeutic nanozymes and future research directions. Adv. Sci..

[30] Wang M., Xu W., Ding Y., Liu X., Fu J., Zhang P. (2025). Nanozymes for regulation of reactive oxygen species in biomedical applications: design, enzyme-like activity and therapeutic mechanisms. Colloids Surf. B Biointerfaces.

[31] Gu Z., Liu X., Qi Z., Fang Z., Jiang Y., Huang Y., Wang Y., Wu L., Yang Y. (2024). An antioxidant nanozyme for targeted cardiac fibrosis therapy post myocardial infarction. J. Nanobiotechnol..

[32] Wang X., Shu C., Wang G., Han P., Zheng L., Xu L., Chen Y. (2025). Recent progress of noble metal-based nanozymes: structural engineering and biomedical applications. Nanoscale.

[33] Touzani A., Ramos-Pan L., Fraga S., Fernandez-Bertolez N., Laffon B., Valdiglesias V. (2025). Systematic review on toxicological effects of platinum nanoparticles: towards their use as safe biomedical tools. Mutat. Res. Rev. Mutat. Res..

[34] Yokel R.A., Hussain S., Garantziotis S., Demokritou P., Castranova V., Cassee F.R. (2014). The yin: an adverse health perspective of nanoceria: uptake, distribution, accumulation, and mechanisms of its toxicity. Environ. Sci. Nano.

[35] Singh N., Savanur M.A., Srivastava S., D'Silva P., Mugesh G. (2019). A manganese oxide nanozyme prevents oxidative damage of biomolecules without affecting the endogenous antioxidant system. Nanoscale.

[36] Li L., Yang X. (2018). The essential element manganese, oxidative stress, and metabolic diseases: links and interactions. Oxid. Med. Cell. Longev..

[37] Li W., Hsiao H.-M., Higashikubo R., Saunders B.T., Bharat A., Goldstein D.R., Krupnick A.S., Gelman A.E., Lavine K.J., Kreisel D. (2016). Heart-resident CCR2+ macrophages promote neutrophil extravasation through TLR9/MyD88/CXCL5 signaling. JCI Insight.

[38] Schloss M.J., Horckmans M., Nitz K., Duchene J., Drechsler M., Bidzhekov K., Scheiermann C., Weber C., Soehnlein O., Steffens S. (2016). The time-of-day of myocardial infarction onset affects healing through oscillations in cardiac neutrophil recruitment. EMBO Mol. Med..

[39] Hou M., Wu X., Zhao Z., Deng Q., Chen Y., Yin L. (2022). Endothelial cell-targeting, ROS-ultrasensitive drug/siRNA co-delivery nanocomplexes mitigate early-stage neutrophil recruitment for the anti-inflammatory treatment of myocardial ischemia reperfusion injury. Acta Biomater..

[40] Fu P., Han X., Lin Q.-Y., Wang N., Sun F.-F., Zhang Y.-L., Li H.-H., Zhang B. (2025). CD11b blockade ameliorates myocardial ischemia/reperfusion injury by reducing neutrophil and monocyte infiltration. J. Am. Heart Assoc..

[41] Hu P., Liu W., Huang J., Su Y., Jiang H., Wu Q., Tao J., Liang S., Lin J., Zheng J. (2025). Navarixin alleviates cardiac remodeling after myocardial infarction by decreasing neutrophil infiltration and the inflammatory response. Front. Pharmacol..

[42] Horckmans M., Ring L., Duchene J., Santovito D., Schloss M.J., Drechsler M., Weber C., Soehnlein O., Steffens S. (2017). Neutrophils orchestrate post-myocardial infarction healing by polarizing macrophages towards a reparative phenotype. Eur. Heart J..

[43] Bao L., Dou G., Tian R., Lv Y., Ding F., Liu S., Zhao R., Zhao L., Zhou J., Weng L., Dong Y., Li B., Liu S., Chen X., Jin Y. (2022). Engineered neutrophil apoptotic bodies ameliorate myocardial infarction by promoting macrophage efferocytosis and inflammation resolution. Bioact. Mater..

[44] Ferraro B., Leoni G., Hinkel R., Ormanns S., Paulin N., Ortega-Gomez A., Sager H.B., Nahrendorf M., Soehnlein O., Steffens S. (2019). Pro-angiogenic macrophage phenotype to promote myocardial repair. J. Am. Coll. Cardiol..

[45] Kolpakov M.A., Guo X., Rafiq K., Vlasenko L., Hooshdaran B., Seqqat R., Wang T., Fan X., Tilley D.G., Kostyak J.C., Kunapuli S.P., Houser S.R., Sabri A. (2020). Loss of protease-activated receptor 4 prevents inflammation resolution and predisposes the heart to cardiac rupture after myocardial infarction. Circulation.

