# Supplementary material for: Neutrophil-tropic APL@Mn3O4 nanozymes alleviate myocardial ischemia-reperfusion injury through redox regulation
Source: Mater Today Bio. 2026 Nov 4;41:103815. doi: 10.1016/j.mtbio.2026.103815 (PMC13769526; doi:10.1016/j.mtbio.2026.103815)
Supplement: Supplementary file 1 [file mmc1.docx]

**Supplementary Material**

**Neutrophil-tropic APL@Mn_3_O_4_ nanozymes alleviate myocardial ischemia-reperfusion injury through redox regulation**

Fu-hang Wang^1^, Yuan-song Zhuang^1^, Jun-jie Ma^1^, Yu-hang Li^2^, Quan-xu An^3^, De-liang Shen^1^*

^1^Department of Cardiology, The First Affiliated Hospital of Zhengzhou University, Zhengzhou 450052, China.

^2^Department of Cardiology, Zhumadian Central Hospital, Zhumadian 463000, China.

^3^Department of Cardiology, Zhoukou Central Hospital, Zhoukou 466000, China.

*Correspondence: De-liang Shen, 18838975868@163.com

**Supplementary Methods**

**S1.1 Analysis of nanoparticle-associated complement component 3 (C3)**

C3 deposition on the nanoparticle formulations was assessed by Western blotting of the nanoparticle-bound protein fraction. APL@Mn_3_O_4_ and L@Mn_3_O_4_ dispersions were adjusted to 50 mg/mL on an Mn_3_O_4_-equivalent basis before serum addition. APL@Mn_3_O_4_ was incubated with normal, heat-inactivated, or cobra venom factor (CVF)-treated mouse serum, whereas L@Mn_3_O_4_ was incubated with normal mouse serum. Each nanoparticle dispersion was mixed with serum at a nanoparticle dispersion-to-serum volume ratio of 1:2, yielding a final Mn_3_O_4_-equivalent concentration of approximately 16.7 mg/mL. The mixtures were incubated at 37 °C for 30 min. The nanoparticles were collected by ultracentrifugation at 60,000 × g for 20 min at 4 °C and washed three times with HEPES buffer to remove unbound serum proteins. The nanoparticle pellets were resuspended in SDS sample buffer, and equal Mn_3_O_4_-equivalent amounts were loaded in each lane. C3 deposited on the nanoparticles was detected using a recombinant rabbit anti-C3 monoclonal antibody (clone EPR19394; Abcam, ab200999; 1:2000; overnight at 4 °C) and an HRP-conjugated secondary antibody, with signals visualized by enhanced chemiluminescence. Band intensities were quantified by densitometry and normalized to the mean of the APL@Mn_3_O_4_ + normal serum group, which was set to 1.

**S1.2 Isolation and culture of primary neonatal rat cardiomyocytes**

Primary neonatal rat cardiomyocytes (NRCMs) were isolated from 1-day-old Sprague–Dawley rats by serial digestion with 0.08% trypsin and 0.08% type II collagenase at 37 °C. The dissociated cells were subjected to differential preplating for 90 min at 37 °C in 5% CO_2_. The non-adherent cardiomyocyte-enriched fraction was collected and cultured in DMEM/F12 supplemented with 10% FBS and 1% penicillin–streptomycin. Experiments were initiated 48 h after plating, when the cells were adherent and beating spontaneously. Cardiomyocyte identity was confirmed by immunofluorescence staining for sarcomeric α-actinin (Abcam, ab9465).

**S1.3 NRCM viability assay**

NRCMs were seeded in 96-well plates at 5 × 10^3^ cells per well and exposed to Mn_3_O_4_ NPs or APL@Mn_3_O_4_ at Mn_3_O_4_-equivalent concentrations ranging from 0 to 200 μg/mL for 24 h. Cell viability was determined using a CCK-8 assay. Absorbance was measured at 450 nm using a microplate reader, and viability was expressed as a percentage of the corresponding untreated control.

**S1.4 Optimization of the neutrophil-to-H9c2 ratio**

To optimize the neutrophil-to-H9c2 ratio, H9c2 cells were seeded in the lower chambers of 12-well Transwell plates at 2 × 10^4^ cells per well and exposed to H_2_O_2_ (150 μM) for 4 h. Neutrophil-to-H9c2 ratios of 0:1, 0.5:1, 1:1, and 2:1 were tested. After 4 h of indirect co-culture, total H9c2 apoptosis was quantified by Annexin V-FITC/PI flow cytometry. The 1:1 ratio was selected for subsequent experiments because it produced a clear but non-saturating apoptotic response.

**S1.5 NRCM indirect co-culture assay**

NRCMs were seeded in the lower chambers at 5 × 10^4^ cells per well and exposed to H_2_O_2_ (150 μM) for 4 h. The H_2_O_2_-containing medium was removed, and the cells were washed twice with PBS before fresh DMEM/RPMI 1640 medium (1:1, v/v) was added. NRCMs were subsequently co-cultured for 4 h with an equal number of activated neutrophils pretreated with PBS, Mn_3_O_4_ NPs, L@Mn_3_O_4_, or APL@Mn_3_O_4_ under the same conditions. NRCM apoptosis was assessed by Annexin V-FITC/PI flow cytometry.

**S1.6 No-primary antibody controls**

For no-primary antibody controls, both primary antibodies were omitted from the CD68/CD86 and CD68/CD206 staining protocols. Myocardial sections were otherwise processed in parallel with the corresponding secondary antibodies and imaged using the same acquisition settings as the experimental samples.

**S1.7 Initial 1-h Transwell migration assay**

The initial migration assay was performed using 3.0-μm-pore Transwell inserts under the same conditions as the 2-h assay described in Section 2.9, except that migration proceeded for 1 h. Tumor necrosis factor-α (TNF-α)-activated neutrophils were pretreated with PBS, Mn_3_O_4_ NPs, L@Mn_3_O_4_, or APL@Mn_3_O_4_, washed three times with cold PBS, resuspended in serum-free medium, and added to the upper chamber. For the negative control, PBS-pretreated neutrophils were used, and the lower chamber contained serum-free medium without fMLP. For all other groups, the lower chamber contained serum-free medium supplemented with fMLP (3 μM). After 1 h, migrated cells were collected from the lower chamber and counted using an automated cell counter.

**S1.8 Tissue manganese quantification by inductively coupled plasma mass spectrometry (ICP-MS)**

For tissue Mn quantification, mice subjected to myocardial ischemia/reperfusion (MI/R) received a single intravenous injection of PBS or APL@Mn_3_O_4_ (5 mg kg^-1^, Mn_3_O_4_-equivalent dose) 1 h after reperfusion. Independent cohorts were analyzed at 24 h, day 7, and day 28 after injection. The heart, liver, spleen, lung, kidney, and brain were harvested, weighed, and digested in trace-metal-grade nitric acid. The digests were diluted with ultrapure water and analyzed using an Agilent 7900 ICP-MS system (Agilent Technologies, Santa Clara, CA, USA). Tissue Mn concentrations were normalized to wet tissue mass and expressed as μg Mn g^-1^ wet tissue.

**S1.9 Biosafety evaluation**

In the MI/R treatment cohorts, blood, liver, spleen, lung, and kidney were collected 48 h after reperfusion from mice receiving PBS, Mn_3_O_4_ NPs, L@Mn_3_O_4_, or APL@Mn_3_O_4_. Serum aspartate aminotransferase (AST) and alanine aminotransferase (ALT) were measured as hepatic function markers, and blood urea nitrogen (BUN) and creatinine (CREA) as renal function markers. Histopathology of the liver, spleen, lung, and kidney was evaluated by hematoxylin and eosin staining.

To assess formulation-associated hematological and systemic inflammatory effects independently of MI/R, healthy male C57BL/6J mice were randomized to receive a single tail-vein injection of PBS or APL@Mn_3_O_4_ (5 mg kg^-1^, Mn_3_O_4_-equivalent dose). Independent cohorts were assessed 48 h and 28 d after injection (n = 6 mice/group/time point). EDTA-anticoagulated whole blood was analyzed with an automated hematology analyzer for red blood cell (RBC), white blood cell (WBC), and platelet (PLT) counts. Serum TNF-α, interleukin-1β (IL-1β), and interleukin-6 (IL-6) were quantified using mouse enzyme-linked immunosorbent assay (ELISA) kits (Elabscience Biotechnology Co., Ltd.; E-EL-M3063, E-EL-M0037, and E-EL-M0044, respectively) according to the manufacturer’s instructions.

**Supplementary Figures**

**Figure S1. Hydrodynamic diameter and zeta potential of Mn_3_O_4_ NPs and APL@Mn_3_O_4_.** (A, B) Hydrodynamic diameters determined by dynamic light scattering (A) and zeta potentials (B) of Mn_3_O_4_ NPs and APL@Mn_3_O_4_. Data are presented as mean ± SD (n = 3 independent nanoparticle preparations).

**Figure S2. Additional physicochemical characterization of Mn_3_O_4_ NPs and APL@Mn_3_O_4_.** (A) Powder X-ray diffraction pattern of Mn_3_O_4_ NPs. Reference reflections for tetragonal hausmannite Mn_3_O_4_ (PDF No. 24-0734) are shown as vertical ticks. (B) X-ray photoelectron spectroscopy survey spectrum of Mn_3_O_4_ NPs. (C–F) High-resolution spectra and peak fitting of Mn 2p (C), Mn 3s (D), O 1s (E), and C 1s (F). (G) Fourier-transform infrared spectra of rat immunoglobulin G (IgG), L@Mn_3_O_4_, and APL@Mn_3_O_4_. The azide-stretching band and IgG-associated amide I and II bands are marked; spectra are vertically offset for clarity. (H, I) Changes in the hydrodynamic diameters of Mn_3_O_4_ NPs (H) and APL@Mn_3_O_4_ (I) over 72 h in PBS at 4 °C or serum at 37 °C. Data in H and I are presented as mean ± SD (n = 3).

**Figure S3. Additional flow-cytometric, complement-associated, and functional analyses of APL@Mn_3_O_4_.** (A) Representative gating of CD45⁺CD11b⁺Ly6G⁺ neutrophils in peripheral blood. (B) Representative gating of Ly6G⁺DiD⁺ neutrophils in the *in vitro* nanoparticle-uptake assay. (C) Immunoblot and densitometric analysis of C3 deposition on APL@Mn_3_O_4_ after incubation with normal, heat-inactivated, or CVF-treated mouse serum and on L@Mn_3_O_4_ after incubation with normal serum. Equal Mn_3_O_4_-equivalent amounts were loaded, and values were normalized to APL@Mn_3_O_4_ + normal serum (= 1). (D) Representative gating for DCFH-DA analysis of bone marrow neutrophils. (E) Absolute neutrophil migration in the initial 1-h Transwell assay. PBS without fMLP served as the negative control; all other groups received fMLP (3 μM). (F) DCFH-DA mean fluorescence intensity in resting (PBS-treated) and TNF-α-activated neutrophils pretreated as indicated. Data in C, E, and F are mean ± SD (n = 6) and were analyzed by one-way ANOVA with Tukey’s multiple-comparisons test. For C, #P < 0.05 versus APL@Mn_3_O_4_ + normal serum. ns, not significant; *P < 0.05, **P < 0.01, ***P < 0.001, and ****P < 0.0001. CVF, cobra venom factor.

**Figure S4. Optimization and quantitative validation of the neutrophil–cardiomyocyte co-culture model.** (A) Representative Annexin V-FITC/PI plots and total apoptosis of H_2_O_2_-injured H9c2 cells at the indicated neutrophil-to-H9c2 ratios. (B–E) DCFH-DA mean fluorescence intensity (B), JC-1 red/green ratio (C), total apoptosis (D), and dead-cell fraction (E) in H_2_O_2_-injured H9c2 cells after co-culture with neutrophils pretreated as indicated. (F) Identification of primary neonatal rat cardiomyocytes (NRCMs) by sarcomeric α-actinin staining (red) and DAPI counterstaining (blue). Scale bars, 50 and 10 μm. (G) NRCM viability after 24-h exposure to Mn_3_O_4_ NPs or APL@Mn_3_O_4_ at the indicated Mn_3_O_4_-equivalent concentrations. (H, I) Quantification (H) and representative Annexin V-FITC/PI plots (I) of NRCM apoptosis under the indicated co-culture conditions. CON denotes H_2_O_2_-injured cardiomyocytes cultured without neutrophils. Data are mean ± SD (n = 6). Statistical analyses were performed using one-way ANOVA with Tukey’s test (A–E and H) or two-way ANOVA with Šídák’s test (G). ns, not significant; *P < 0.05, **P < 0.01, ***P < 0.001, and ****P < 0.0001.

**Figure S5. Acute myocardial injury and short-term systemic tolerability following myocardial ischemia/reperfusion.** (A) Quantification of the area at risk relative to the left ventricular area (AAR/LV) and the infarct area relative to the AAR using Evans blue/TTC staining. (B) Mean fluorescence intensity of myocardial dihydroethidium (DHE) staining. (C) Representative no-primary antibody controls for CD68/CD206 and CD68/CD86 immunofluorescence staining with DAPI counterstaining (scale bars, 50 μm). (D) Serum aspartate aminotransferase (AST), alanine aminotransferase (ALT), blood urea nitrogen (BUN), and creatinine (CREA) levels at 48 h after reperfusion. (E) Representative hematoxylin and eosin (H&E)-stained sections of the liver, spleen, lung, and kidney at 48 h after reperfusion (scale bars, 200 μm). Data in A, B, and D are presented as mean ± SD (n = 6). Each outcome in A, B, and D was analyzed by one-way ANOVA followed by Tukey’s multiple-comparisons test. ns, not significant; *P < 0.05, **P < 0.01, ***P < 0.001, and ****P < 0.0001.

**Figure S6. Hematological and systemic inflammatory indices and time-dependent tissue Mn concentrations after APL@Mn_3_O_4_ administration.** (A–C) Red blood cell (RBC), white blood cell (WBC), and platelet (PLT) counts in healthy mice at 48 h and day 28 after a single intravenous injection of PBS or APL@Mn_3_O_4_. (D–F) Serum TNF-α, IL-1β, and IL-6 concentrations in the healthy-mouse groups at the same time points. (G–L) ICP-MS quantification of Mn in the heart (G), liver (H), spleen (I), lung (J), kidney (K), and brain (L) of MI/R mice at 24 h, day 7, and day 28 after the same treatments. Data are presented as mean ± SD (n = 6 mice/group/time point). Each panel was analyzed by ordinary two-way ANOVA with Šídák’s multiple-comparisons test comparing PBS with APL@Mn_3_O_4_ at each time point. ns, not significant; #P < 0.05 versus PBS (G–L).
